# The first WHO global survey on infection prevention and control in health-care facilities

**DOI:** 10.1016/S1473-3099(21)00809-4

**Published:** 2022-06

**Authors:** Sara Tomczyk, Anthony Twyman, Marlieke E A de Kraker, Ana Paula Coutinho Rehse, Ermira Tartari, João Paulo Toledo, Alessandro Cassini, Didier Pittet, Benedetta Allegranzi

**Affiliations:** a Infection Prevention and Control Technical and Clinical Hub, Department of Integrated Health Services, WHO, Geneva, Switzerland; b Department for Infectious Disease Epidemiology, Robert Koch Institute, Berlin, Germany; c Infection Control Programme, University of Geneva Hospitals and Faculty of Medicine, Geneva, Switzerland; d Infectious Hazard Management Programme, Health Emergencies Programme, WHO Regional Office for Europe, Copenhagen, Denmark; e Faculty of Health Sciences, University of Malta, Msida, Malta; f Clinical Management of Infectious Diseases, Health Emergencies Department, Pan American Health Organization, Washington, DC, USA

## Abstract

**Background:**

WHO core components for infection prevention and control (IPC) are important building blocks for effective IPC programmes. To our knowledge, we did the first WHO global survey to assess implementation of these programmes in health-care facilities.

**Methods:**

In this cross-sectional survey, IPC professionals were invited through global outreach and national coordinated efforts to complete the online WHO IPC assessment framework (IPCAF). The survey was created in English and was then translated into ten languages: Arabic, Chinese, English, French, German, Italian, Japanese, Russian, Spanish, and Thai. Post-stratification weighting was applied and countries with low response rates were excluded to improve representativeness. Weighted median scores and IQRs as well as weighted proportions (Nw) meeting defined IPCAF minimum requirements were reported. Indicators associated with the IPCAF score were assessed using a generalised estimating equation.

**Findings:**

From Jan 16 to Dec 31, 2019, 4440 responses were received from 81 countries. The overall weighted IPCAF median score indicated an advanced level of implementation (605, IQR 450·4–705·0), but significantly lower scores were found in low-income (385, 279·7–442·9) and lower-middle-income countries (500·4, 345·0–657·5), and public facilities (515, 385–637·8). Core component 8 (built environment; 90·0, IQR 75·0–100·0) and core component 2 (guidelines; 87·5, 70·0–97·5) scored the highest, and core component 7 (workload, staffing, and bed occupancy; 70·0, 50–90) and core component 3 (education and training; 70 ·0, 50·0–85·0) scored the lowest. Overall, only 15·2% (Nw: 588 of 3873) of facilities met all IPCAF minimum requirements, ranging from 0% (0 of 417) in low-income countries to 25·6% (278 of 1087) in primary facilities, 9% (24 of 268) in secondary facilities, and 19% (18 of 95) in tertiary facilities in high-income countries.

**Interpretation:**

Despite an overall high IPCAF score globally, important gaps in IPC facility implementation and core components across income levels hinder IPC progress. Increased support for more effective and sustainable IPC programmes is crucial to reduce risks posed by outbreaks to global health security and to ensure patient and health worker safety.

**Funding:**

WHO and the Infection Control Programme, University of Geneva Hospitals and Faculty of Medicine.

**Translations:**

For the French and Spanish translations of the abstract see Supplementary Materials section.

## Introduction

The COVID-19 pandemic has emphasised the crucial role of infection prevention and control (IPC) programmes and practices to ensure the safety of patients and health workers through preparedness and response to outbreaks. It has also shown that even advanced health systems have deficiencies in their implementation of IPC practices and preparedness to outbreaks.^1^ Effective IPC programmes are the foundation for reducing endemic health-care-associated infections (HAIs), the spread of antimicrobial resistance (AMR), and for the containment of emerging pathogens, thus contributing to quality of care as an essential component of universal health coverage.^2^

Over the past decade, WHO and other international agencies have demonstrated the global burden of HAIs and AMR, with an urgent call to action to improve IPC.^3^, ^4^, ^5^ In the EU, a population-based modelling study estimated that six types of HAIs accounted for about double the total burden in morbidity and deaths than all other 32 reported communicable diseases combined.^6^ Health-care facilities are often the starting point or multiplier for infectious disease outbreaks, which can contribute to further transmission in the community. Unprepared health systems are unable to withstand the shock of an outbreak, as shown by the west African 2014–16 Ebola outbreak and the COVID-19 pandemic.^7^ Importantly, evidence-based IPC interventions have been shown to be effective in preventing at least 50% of HAIs.^8^ A report by the Organisation for Economic Co-operation and Development found that the most cost-saving interventions to reduce AMR in health care were those aimed at combining hospital hygiene and antimicrobial stewardship improvement, and had the potential to prevent three of four attributable deaths.^9^

**Research in context**

**Evidence before this study**

Infection prevention and control (IPC) programmes and practices play a vital role to ensure outbreak preparedness and control, including patient safety and quality of care, which remain essential components of universal health coverage across health systems worldwide. However, detailed IPC evaluations using standardised validated tools, such as the WHO IPC self-assessment framework (IPCAF), are limited. We searched *PubMed, medRxiv, bioRxiv, arXiv*, and WHO global health databases between Jan 1, 2011, and June 30, 2021, for peer-reviewed and preprint articles in English, French, and Spanish that reported global assessments of IPC programme implementation at the facility level using the search terms “infection control” and “global survey”. We identified four studies using the IPCAF in a comparable manner in Austria, Germany, Ghana, and Pakistan. Results varied by income level. Although median scores in Austria and Germany reflected advanced levels of performance, those in Ghana and Pakistan reflected levels ranging from intermediate to basic or inadequate. Additionally, a national study in Georgia used an adapted version of the IPCAF; a systematic review of studies in mainland China described the assessment of IPC implementation according to the WHO core components; and a study in Kenya assessed facilities using the water, sanitation, and hygiene facility improvement tool. Most other identified studies focused on hand hygiene programme implementation, as well as needs, access, and availability of individual IPC elements at the country level.

**Added value of this study**

This study reports findings from the first WHO global survey assessing IPC programme implementation at the facility level using IPCAF, a validated and standardised tool to assess the WHO IPC core components, which represent the global gold standard, evidence-based, recommendations for IPC at the national and health-care facility level. Using robust methodology, this study provides a global snapshot of IPC programme implementation across all six WHO regions (Africa, Americas, Eastern Mediterranean, Europe, South-East Asia, and the Western Pacific) and World Bank income levels. With a total of 4440 responses from 81 countries, we found a total weighted IPCAF median score of 605 (IQR 450·4–705·0), which corresponded to an advanced level of performance. However, significantly lower scores were observed in low-income and lower-middle-income countries and in public health-care facilities. Overall, core component 8 (built environment, materials, and equipment for IPC; 90, IQR 75·0–100·0) and core component 2 (IPC guidelines; 87·5, 70·0–97·5) had the highest score, and core component 7 (workload, staffing, and bed occupancy; 70·0, 50·0–90·0) and core component 3 (IPC education and training; 70·0, 50·0–85·0) had the lowest scores. Among low-income countries, core component 4 (health-care-associated infection [HAI] surveillance) and core component 6 (monitoring, audit of IPC practices and feedback) had the lowest scores. No health-care facility in low-income countries met all IPCAF minimum requirements compared with the 25·6% in primary health-care facilities, 9% in secondary facilities, and 18·9% of tertiary facilities in high-income countries. The survey also included a parallel hand hygiene component using another dedicated standardised assessment tool measuring the implementation of the WHO hand hygiene improvement strategy. We found a strong correlation between selected components of the two tools, demonstrating their alignment and consistency in health-care facility self-assessment and emphasising hand hygiene as an important predictor of the overall IPC level.

**Implications of all the available evidence**

Overall, health-care facilities had an advanced level of IPC implementation, but this varied across income levels. Although most facilities reported having an IPC programme, few met all IPC minimum requirements recommended by WHO. Efforts to support the long-term development of IPC programmes and stepwise improvement are crucial, particularly in low-income and middle-income countries, which remain the most vulnerable. Our findings show that further investments are needed in all countries to improve the effective implementation of IPC training programmes and meet adequate workload and staffing requirements, as well as standards for bed occupancy and spacing between beds. In low-resource settings, efforts are particularly needed to improve HAI surveillance and IPC monitoring and feedback. Although some of these investments might have been made after this survey to combat the COVID-19 pandemic, ensuring sustained long-term implementation through stronger IPC policies and regulations, a competent and adequate workforce, and leadership support at the highest levels through dedicated budgets and accountability mechanisms is crucial.

On the basis of systematic reviews and expert consensus, the WHO guidelines on core components of IPC programmes have become a global reference for building effective IPC programmes at the national and acute care health facility level.^2^ In 2018, WHO launched a validated IPC assessment framework (IPCAF) to support the implementation of the core components at the acute care level.^10^ The usability and reliability of the IPCAF was tested in a global study.^11^ Notably, the use of IPCAF and its scoring system allows for the provision of a baseline situational analysis within a health-care facility and measures progress over time by identifying gaps and strengths, according to a step-wise approach to improvement.^12^ In 2019, WHO further specified the minimum requirements for IPC programmes, with the aim of supporting the stepwise implementation towards achievement of the full set of core components.^13^ These requirements represent the starting point to build and sustain strong and effective IPC programmes at the national and facility level, including the basic IPC standards that should be in place to provide minimum protection and safety to patients, health workers, and visitors.

To date, evaluations of IPC programmes using standardised tools have been limited to local or country assessments in mainly high-income countries.^14^, ^15^, ^16^ We present here the first large-scale survey using IPCAF to assess the global situation of IPC programme implementation at the health-care facility level and provide a baseline that can be used for future monitoring and evaluation to document progress and inform IPC improvement efforts. We aimed to identify differences in implementation across regions, income levels, and types of health-care facilities. The survey also included a parallel hand hygiene component using another dedicated standardised assessment tool.^17^ Accordingly, we investigate the correlation between selected IPC indicators and hand hygiene indicators.

## Methods

### IPCAF survey instrument

IPCAF is a structured, closed-formatted questionnaire with an associated scoring system and is usually self-administered (appendix 3 pp 2–28).^10^ It comprises 81 indicators subdivided into eight sections corresponding to the eight WHO IPC core components: (1) IPC programme; (2) IPC guidelines; (3) IPC education and training; (4) HAI surveillance; (5) multimodal strategies; (6) monitoring and audit of IPC practices and feedback; (7) workload, staffing, and bed occupancy; and (8) the built environment, and materials and equipment for IPC. Each section generates a score between 0 and 100. According to the final score (ranging from 0 to 800), the facility IPC programme implementation is categorised into four levels: inadequate (0–200), basic (201–400), intermediate (401–600), or advanced (601–800).

### Study design and participants

WHO did a global, cross-sectional survey among health-care facilities consisting of two survey instruments: (1) IPCAF, and (2) the hand hygiene self-assessment framework (HHSAF) as described elsewhere.^17^ The survey was created in English and translated into ten languages: Arabic, Chinese, English, French, German, Italian, Japanese, Russian, Spanish, and Thai. Data were collected using LimeSurvey, an online password-protected survey platform with built-in validity checks. The platform was pilot-tested among 20 participants from low-income and high-income countries in December, 2018.^18^ Training materials and instructions for survey completion were provided (appendix 3 pp 2–28). IPC professionals, in collaboration with other colleagues, were asked to complete the survey, including questions on their professional background and facility demographics.

Because national health-care facility registries were not systematically available across all countries, it was not possible to do multistage sampling and a convenience sample of health-care facilities was collected. To aim for global representativeness, we proportionally stratified a target sample of countries (n=84) by WHO region and income level and tracked health-care facility enrolment by these targets as part of recruitment efforts.^19^, ^20^ The sample was based on an expected proportion of 37% of countries reporting the presence of an IPC programme in all health-care facilities among the 194 member states and 80% confidence. This expected proportion was estimated according to the results of the Tripartite AMR Country Self-assessment Survey (TrACSS),^21^ in which countries report their annual progress of national AMR action plan implementation, including an indicator on IPC programme implementation—namely, no IPC programme implementation (levels A+B) compared with IPC programme implementation in selected health-care facilities (level C) or nationwide (levels D+E).^21^

The study was approved by the WHO Ethics Review Committee (#0003127). As the survey was a facility-based assessment and did not include individual patient or health worker data, consent was not applicable. However, instructions before survey agreement included information on data use and confidentiality (appendix 3 pp 2–3). All facility data were kept confidential, and access was restricted to the research team at the WHO IPC Technical and Clinical Hub and the Infection Control Programme at the University of Geneva Hospitals.

### Procedures

Health-care facilities were invited to participate through email by the global WHO IPC unit (database of 22 144 health-care facilities from 182 WHO member states registered with the SAVE LIVES: Clean Your Hands campaign). The survey was also promoted through the WHO website, newsletters and social media, the WHO Global IPC Network, the WHO Partnership for Patient Safety, and conferences. WHO regional and country offices were directly approached to promote national participation and 43 ministries of health or other national bodies expressed interest in centrally coordinating data collection from health-care facilities in their own country using a country-specific link (appendix 3 pp 29–30).

After data cleaning, three selection steps were applied to survey responses (appendix 3 pp 29–30). In step one, IPCAF submissions were restricted to those with at least one fully completed IPC core components section. In step two, a systematic approach was used to de-duplicate health-care facilities with multiple survey responses from the same facility using a geospatial clustering algorithm (appendix 3 pp 29–31). In step three, a minimal threshold excluded responses from countries with a ratio of number of survey responses per capita in the lowest ventile (appendix 3 pp 29–31). To further improve global representativeness, post-stratification weights were applied on the basis of a ranking algorithm using country, World Bank country income level,^19^ WHO region,^20^ facility care level (primary, secondary, or tertiary), and type of facility (private or public; appendix 3 p 31).

The primary study outcomes were the overall and core component-specific IPCAF scores measuring the level of IPC implementation in health-care facilities. Secondary study outcomes were the proportion of health-care facilities fufilling the WHO IPC minimum requirements, and the correlation of component-specific IPCAF and element-specific HHSAF scores.

### Statistical analysis

Data describing the characteristics of survey respondents are presented as absolute frequencies and proportions. Weighted IPCAF score medians and IQRs were reported overall (restricted to only fully completed surveys) and for each core component (restricted to surveys completed for the respective core component), including by WHO region and World Bank income levels. Additionally, IPCAF scores were reported according to country status in the 2019–20 TrACSS. To assess the association between facility characteristics and the total IPCAF score, a multivariable, generalised estimating equation model with robust standard errors was used to account for clustering at the country level and post-stratification weights (R package “geepack”, function “geeglm”). The Box-Cox log-likelihood test was used to assess appropriateness of linear regression.

Selected IPCAF indicators were specified by WHO as minimum requirements for IPC programmes (appendix 3 pp 29–31).^13^ Weighted frequencies (Nw) of health-care facilities meeting the individual minimum requirements were summarised by income and facility care levels. As the minimum requirements are indicated separately by WHO for primary, secondary, and tertiary health-care facilities, this analysis was restricted to facilities reporting the care level (ie “other” level of care was excluded, such as specialised or unspecified centres) and fully completing the survey or respective core component. A subset analysis according to country income level and facility care level was done among complete submissions for which the care level was specified to assess the weighted frequencies of facilities meeting the individual IPCAF indicator responses that fulfilled WHO IPC minimum requirements.^13^ The correlation of specific corresponding, component-specific IPCAF and element-specific HHSAF scores were assessed using a weighted Pearson correlation coefficient (R^2^) with 95% CIs among completed responses for health-care facilities responding to both surveys. p<0·05 was considered statistically significant. Missing data were addressed by restricting the respective analyses to fully completed surveys overall or by specific components. All analyses were done using R statistical software version 3.6.1.

### Role of the funding source

Authors from the funding body WHO were involved in study design, data collection, data analysis, data interpretation, and writing of the report.

## Results

**Figure 1:**
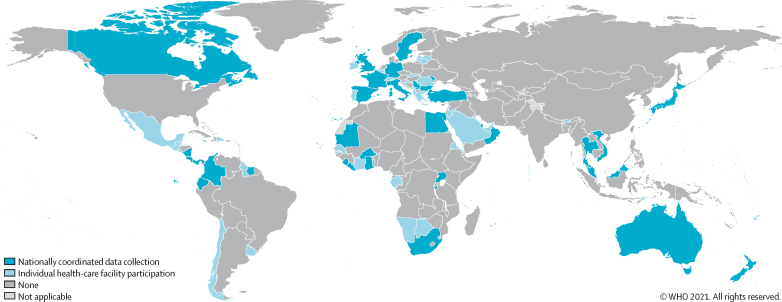
Country participation in the Infection Prevention and Control Assessment Framework global survey 2019, after application of the minimal response threshold Total number of countries=81. Total number with nationally coordinated data collection=37. The designations employed and the presentation of the material in this publication do not imply the expression of any opinion whatsoever on the part of WHO concerning the legal status of any country, territory, city or area or of its authorities, or concerning the delimitation of its frontiers or boundaries. Dotted and dashed lines on maps represent approximate border lines for which there may not yet be full agreement. Published with permission of the WHO GIS Centre for Health, DNA/DDI.

From Jan 16 to Dec 31, 2019, 4673 unique responses were received from 126 countries (65%) of 194 WHO member states (appendix 3 pp 29–30). Country participation ranged from one to 499 responses per country (ratio of responses per capita from 0·001 per 100 000 to 6·683 per 100 000). After applying the threshold to improve global representativeness (responses per capita <0·026 per 100 000 were excluded), 4440 responses from 81 countries were retained (42% of WHO member states; table 1; appendix 3 pp 29–30). Stratification by WHO region was: region of the Americas, 60% (21 of 35 countries); European region, 43% (23 of 53 countries); Eastern Mediterranean region, 43% (nine of 21 countries); South-East Asia region, 36% (four of 11 countries); African region, 34% (16 of 47 countries); and the Western Pacific region, 30% (eight of 27 countries). A greater proportion of countries from high-income (55%, 34 of 62) and upper-middle-income (52%, 28 of 54) levels participated than those in lower-middle-income (27%, 13 of 49) and low-income (21%, six of 29) levels (figure 1).

**Table 1:** Characteristics of survey responses in the WHO global survey of IPC programmes, 2019*

	**All selected responses (n=4440)**
**WHO regions**	
African	698 (15·7%)
Eastern Mediterranean	523 (11·8%)
Europe	1393 (31·4%)
Americas	557 (12·5%)
South-East Asia	517 (11·6%)
Western Pacific	752 (16·9%)
**World Bank income level**	
Low-income	173 (3·9%)
Lower-middle-income	728 (16·4%)
Upper-middle-income	1638 (36·9%)
High-income	1901 (42·8%)
**Participation in nationally coordinated data collection**	
Yes	1751 (39·4%)
No	2689 (60·6%)
**Facility type**	
Public	2554 (57·5%)
Private	689 (15·5%)
Other†	1197 (27·0%)
**Facility level of care**	
Primary	1419 (32·0%)
Secondary	879 (19·8%)
Tertiary	702 (15·8%)
Other‡	1440 (32·4%)

Data are n (%). IPCAF=Infection Prevention and Control Assessment Framework.

* n=4440, but 4192 (94·4%) were fully completed for all IPCAF core components.

† Includes mixed private-public health-care facilities, mission hospitals, or unspecified.

‡ Includes specialised centres (cardiology, HIV, nephrology, neurology, oncology, obstetrics, plastic surgery, palliative care, rehabilitation, tuberculosis, or unspecified).

Results were weighted for country, region, income level, facility type, and care level. The total weighted IPCAF median score was 605·0 (IQR 450·4–705·0), which is close to the lower range of the advanced IPC implementation level (score 600–800). Overall, 50·7% of health-care facilities attained an advanced IPCAF level (Nw: 2074 of 4087), 29·8% attained an intermediate level (Nw: 1216 of 4087), 17·5% attained a basic level (Nw: 715 of 4087), and 2·0% attained an inadequate level (Nw: 82 of 4087). The weighted median score varied across income levels from 385·0 (IQR 279·7–442·9; basic level) in low-income countries to 657·5 (562·5–717·5; advanced level) in high-income countries. Similarly, the total score varied according to type of health-care facility, ranging from 515·0 (IQR 385·0–637·8; intermediate level) in public facilities to 675·0 (542·1–730·0; advanced level) in private facilities (table 2). Health-care facilities from countries with the most advanced levels of IPC programme implementation according to the 2019–20 TrACSS indicators had a higher total IPCAF median score (637·5, IQR 515·0–710·0) than did those with no IPC programme implementation reported to TrACSS (477·5, 322·2–650·0**;** table 2).

**Table 2:** Weighted IPCAF scores overall and by selected factors*

	**Core component 1, IPC programme**		**Core component 2, IPC guidelines**		**Core component 3, IPC education**		**Core component 4, HAI surveillance**		**Core component 5, multimodal**		**Core component 6, monitoring and feedback**		**Core component 7, workload, staffing, and bed occupancy**		**Core component 8, built environment**		**Total score**	
	N	Weighted median (IQR)	N	Weighted median (IQR)	N	Weighted median (IQR)	N	Weighted median (IQR)	N	Weighted median (IQR)	N	Weighted median (IQR)	N	Weighted median (IQR)	N	Weighted median (IQR)	N	Weighted median (IQR)
Overall	4407	77·5 (57·5–90·0)	4368	87·5 (70·0–97·5)	4396	70·0 (50·0–85·0)	4331	77·5 (47·5–92·5)	4383	75·0 (45·0–85·0)	4387	72·5 (52·5–90·0)	4378	70·0 (50·0–90·0)	4347	90·0 (75·0–100·0)	4192	605·0 (450·4–705·0)
Region																		
Africa	685	55·0 (40·0–77·5)	663	67·5 (42·5–90·0)	673	57·9 (30·0–78·8)	653	32·3 (2·5–65·0)	675	60·5 (30·0–85·0)	682	50·0 (30·0–82·5)	678	60·0 (35·0–80·0)	665	75·0 (60·0–93·4)	595	415·3 (290·0–581·7)
Eastern Mediterranean	522	95·0 (82·5–100·0)	522	97·5 (90·0–100·0)	521	90·0 (70·0–95·0)	520	87·5 (75·0–100·0)	519	85·0 (80·0–95·0)	519	82·5 (70·0–90·0)	518	90·0 (70·0–100·0)	518	95·0 (90·0–100·0)	514	715·0 (632·5–740·0)
Europe	1388	82·5 (70·0–90·0)	1376	92·5 (77·5–100·0)	1388	75·0 (60·0–85·0)	1367	87·5 (71·4–95·0)	1378	75·0 (44·9–85·0)	1375	80·0 (67·5–90·0)	1374	80·0 (70·0–95·0)	1373	95·0 (90·0–100·0)	1339	650·0 (558·6–720·3)
Americas	553	75·0 (60·0–84·1)	548	85·0 (77·5–95·0)	552	65·0 (45·0–80·0)	544	80·0 (67·5–90·0)	549	65·0 (45·0–80·0)	550	70·0 (51·1–85·0)	548	70·0 (46·1–80·0)	542	82·5 (67·5–95·0)	531	567·5 (477·5–639·3)
South-East Asia	514	42·5 (27·5–77·5)	516	72·5 (42·5–85·0)	516	45·0 (15·0–70·0)	504	45·0 (22·5–81·3)	516	45·0 (20·0–75·0)	516	62·5 (35·0–87·5)	516	60·0 (35·0–70·0)	515	70·0 (67·5–87·5)	500	425 (280·0–657·5)
Western Pacific	745	75·0 (62·5–85·0)	743	90·0 (70·7–100·0)	746	75·0 (60·0–85·0)	743	67·9 (65·0–87·5)	746	70·0 (50·0–86·5)	745	75·0 (57·5–90·0)	744	72·9 (49·8–95·0)	734	95·0 (85·0–100·0)	713	636·0 (521·4–698·5)
Income level																		
Low-income	172	50·0 (13·3–62·9)	172	60·0 (27·5–77·5)	171	50·0 (23·8–60·0)	169	12·5 (0–32·5)	171	47·4 (30·0–75)	172	37·5 (27·5–55·0)	173	55·0 (35·0–75·0)	172	70·0 (57·5–80·0)	167	385·0 (279·7–442·9)
Lower-middle-income	724	60·6 (42·5–80·0)	725	80·0 (55·0–95·0)	723	65·0 (35·0–80·0)	720	62·5 (30·0–90·0)	719	60·0 (20·0–85·0)	719	70·0 (35·0–90·0)	720	60·0 (35·0–80·8)	719	75·0 (67·5–90·0)	711	500·4 (345·0–657·5)
Upper-middle-income	1624	80·0 (62·5–92·5)	1601	87·5 (72·5–97·5)	1613	70·0 (55–90)	1581	82·5 (60–95)	1613	75·0 (35–90)	1617	75·0 (55–92·3)	1610	70·0 (58·8–90)	1595	92·5 (80–97·5)	1511	632·5 (482·5–710)
High-income	1887	82·5 (72·5–92·5)	1870	92·5 (85·0–100·0)	1889	75·0 (60·0–90·0)	1861	85·0 (70·0–92·5)	1880	80·0 (60·0–90·0)	1879	80·0 (62·5–87·5)	1875	80·0 (65·0–95·0)	1861	95·0 (90·0–100·0)	1803	657·5 (562·5–717·5)
Participation in nationally coordinated data collection																		
Yes	1743	72·5 (50·0–85·1)	1737	82·5 (67·3–97·5)	1740	65·0 (55·0–80·0)	1717	70·0 (30·2–87·5)	1742	60·0 (40·0–80·0)	1742	67·5 (47·5–82·5)	1741	70·0 (55·0–85·0)	1728	87·5 (65·0–96·3)	1683	553·9 (445·0–647·5)
No	2664	80·0 (60·0–90·0)	2631	90·0 (70·0–97·5)	2656	70·0 (50–85)	2614	80·0 (52·5–92·5)	2641	75·0 (45·0–90·0)	2645	75·0 (55·0–90·0)	2637	74·4 (50–90)	2619	92·5 (75–100)	2509	622·5 (452·3–712·2)
Facility type																		
Public	2530	70·0 (47·5–82·5)	2502	80·0 (65·0–92·5)	2527	65·0 (45·0–80·0)	2482	67·5 (32·5–85·0)	2509	60·0 (30·0–80·0)	2515	67·5 (37·5–82·5)	2507	60·0 (39·5–75·0)	2485	80·0 (65–95)	2397	515·0 (385–637·8)
Private	683	82·5 (70·0–92·5)	671	90·7 (77·5–100·0)	673	75·0 (60·0–90·0)	664	87·5 (65·0–95·0)	679	80·0 (60·0–90·0)	676	80·0 (60·0–90·5)	675	85·0 (70·0–98·0)	666	95·0 (87·5–100·0)	615	675·0 (542·1–730·0)
Other†	1194	90·0 (70·0–95·0)	1195	95·0 (82·5–100·0)	1196	85·0 (70·0–95·0)	1185	85·0 (70·0–97·5)	1195	65·0 (30·2–90·0)	1196	85·0 (62·5–95·0)	1196	80·0 (60·0–90·0)	1196	95·0 (87·5–100·0)	1180	650·0 (570·8–732·5)
Facility level of care																		
Primary	1405	75·0 (55·0–87·5)	1392	87·5 (70·0–97·5)	1402	70·0 (50·0–85·0)	1383	75·0 (45·0–90·0)	1398	70·0 (45·0–85·0)	1404	72·5 (50·0–90·0)	1395	70·0 (50·0–90·0)	1388	90·0 (72·5–100·0)	1333	601·0 (435·0–699·4)
Secondary	876	77·5 (65·0–90·0)	863	72·5 (55·0–95·0)	868	65·0 (40·0–75·0)	857	67·5 (40·0–90·0)	866	65·0 (45·0–85·0)	866	67·5 (37·5–82·5)	865	70·0 (50·0–85·0)	859	92·5 (82·5–100·0)	830	562·5 (462·5–657·5)
Tertiary	692	87·5 (62·5–95·0)	686	87·5 (77·5–100·0)	694	70·0 (55·8–80·0)	680	87·5 (65·0–95·0)	687	75·0 (35·0–90·0)	687	85·0 (72·5–90·0)	687	70·0 (40·0–85·0)	675	95·0 (75·0–100·0)	645	660·0 (515·0–727·5)
Other‡	1434	90·0 (82·5–95·0)	1427	95·0 (92·5–100·0)	1432	85·0 (75·0–95·0)	1411	95·0 (85·0–97·5)	1432	85·0 (65·0–95·0)	1430	87·5 (75·0–95·0)	1431	90·0 (75·0–95·0)	1425	100·0 (95·0–100·0)	1384	712·5 (637·5–762·5)
Annual country status in TrACSS monitoring of national AMR action plans§																		
IPC programme implementation in selected facilities or nationwide	3028	80·0 (65·0–95·0)	3018	90·0 (77·5–100·0)	3031	75·0 (60·0–85·0)	2994	82·5 (62·5–92·5)	3018	80·0 (55·0–90·0)	3017	75·0 (57·5–87·5)	3014	75·0 (55·0–90·0)	3002	92·5 (82·5–100·0)	2931	637·5 (515·0–710·0)
No IPC programme implementation	997	62·5 (37·5–82·5)	971	72·5 (35·0–97·5)	984	55·0 (25·0–75·0)	960	55·0 (37·5–80·2)	986	45·0 (10·0–75·0)	989	67·5 (35·0–87·5)	983	65·0 (40·0–80·0)	968	87·5 (70·0–95·0)	893	477·5 (322·5–650·0)
Missing	382	77·5 (52·5–87·5)	379	85·0 (67·5–95·0)	381	70·0 (45·0–85·0)	377	82·5 (50·0–95·0)	379	70·0 (40·0–90·0)	381	75·0 (37·5–95·0)	381	75·0 (50·0–95·0)	377	85·0 (67·5–100·0)	368	575·0 (435·0–737·8)

Data are n, or weighted median (IQR). IPCAF=Infection Prevention and Control Assessment Framework. HAI=health-care-associated infection. TrACSS=Tripartite AMR country self-assessment survey. AMR=antimicrobial resistance.

* Includes per component all facilitiçes that completed that survey part.

† Includes mixed private-public health-care facilities, mission hospitals, or unspecified.

‡ Includes specialised centres (cardiology, HIV, nephrology, neurology, oncology, obstetrics, plastic surgery, palliative care, rehabilitation, tuberculosis, or unspecified).

§ 2019–20 TrACSS administered by WHO. In the TrACSS, countries report annual progress on national AMR action plan implementation.

Weighted multivariable regression analysis showed that a lower total IPCAF score was significantly associated with low-income (−229·8 points lower [95% CI −352·3 to −107·3]) and lower-middle-income (−80·1 points lower [–148·1 to −12·1]) country status than high-income country status. A higher total IPCAF score was significantly associated with health-care facilities reporting their care level as Other (98·7 points higher [95% CI 36·7 to 160·6]) and categorised as a tertiary care facility (73 points higher [23·3 to 122·8]) rather than a primary care facility. Similarly, a private facility type was associated with an increase in the total IPCAF score (85 points higher [95% CI 40·6 to 129·5]) compared with a public facility. WHO region and nationally coordinated data collection were not independent predictors of the overall IPCAF score (table 3).

**Table 3:** Factors associated with overall IPCAF score*

	**Coefficient**†**(95% CI)**
**Income level**	
High-income	1 (ref)
Upper-middle-income	−28·3 (−85·2 to 28·7)
Lower-middle-income	−80·1 (−148·1 to −12·1)‡
Low-income	−229·8 (−352·3 to −107·3)‡
**Region**	
Africa	1 (ref)
Americas	−1·8 (−90·5 to 87)
Eastern Mediterranean	80·5 (−23·8 to 184·8)
Europe	10·5 (−88 to 108·8)
South-East Asia	−101·5 (−238·5 to 35·6)
Western Pacific	9·2 (−106·3 to 124·7)
**Participation in nationally coordinated data collection**	
No	1 (ref)
Yes	−8·9 (−44·0 to 26·1)
**Facility level of care**	
Primary	1 (ref)
Secondary	−28·3 (−83·9 to 27·2)
Tertiary	73 (23·3 to 122·8)‡
Other§	98·7 (36·7 to 160·6)‡
**Facility type**	
Public	1 (ref)
Private	85 (40·6 to 129·5)‡
Other¶	38·2 (−50 to 126·5)

IPCAF=Infection Prevention and Control Assessment Framework.

* Includes only complete surveys (n=4192).

† A weighted multivariate random intercepts linear regression model (generalised estimating equation) was used. Post-stratification weights were included and country added as a random intercept.

‡ Significantly different *vs* reference category.

§ Includes mixed private-public health-care facilities, mission hospitals, or unspecified.

¶ Includes specialised centres (cardiology, HIV, nephrology, neurology, oncology, obstetrics, plastic surgery, palliative care, rehabilitation, tuberculosis, or unspecified).

**Figure 2:**
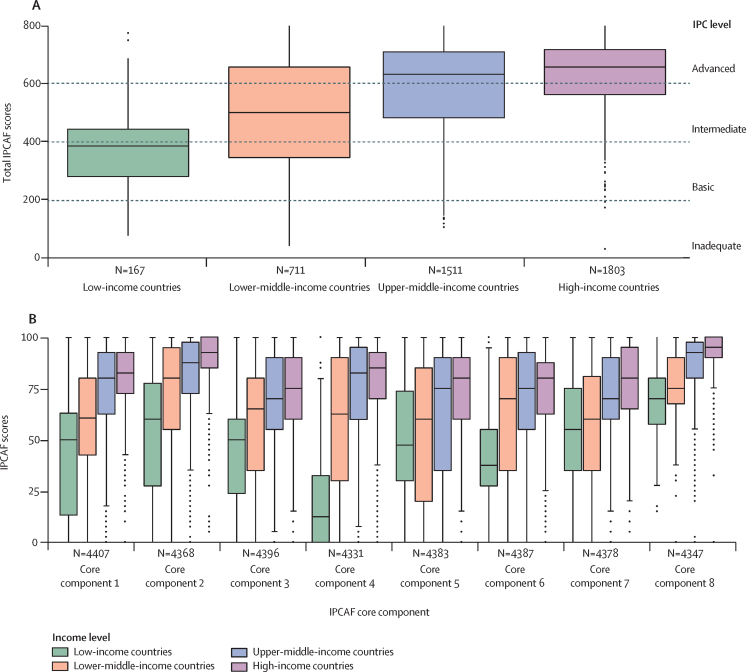
Weighted IPCAF overall and core component-specific scores by income level (A) Weighted overall scores, only includes complete surveys (n=4192). (B) Core-component-specific scores, includes per component all health-care facilities that completed corresponding survey section. The boxes represent the IQR, including the median which is represented by the middle horizontal line. The whiskers represent the full range and the dots represent the outliers. IPCAF=Infection Prevention and Control Assessment Framework. Core component 1=IPC programme. Core component 2=IPC guidelines. Core component 3=IPC education and training. Core component 4=health-care-associated infection surveillance. Core component 5=multimodal strategies. Core component 6=monitoring, audit of IPC practices and feedback. Core component 7=workload, staffing, and bed occupancy. Core component 8=built environment, materials, and equipment for IPC.

Among the weighted, median core component-specific IPCAF scores, core component 8 (built environment, materials, and equipment for IPC; 90·0, IQR 75·0–100·0) and core component 2 (IPC guidelines; 87·5, 70·0–97·5) scored the highest. Core component 7 (workload, staffing, and bed occupancy; 70·0, IQR 50·0–90·0) and core component 3 (IPC education and training; 70·0, 50·0–85·0) scored the lowest. Weighted median scores for the remaining core components scored between 72·5 and 77·5 (table 2). The largest differences between low-income and high-income countries were for core component 4 (HAI surveillance; 12·5 [IQR 0–32·5] *vs* 85 [70·0–92·5]), core component 6 (monitoring, audit of IPC practices and feedback; 37·5 [27·5–55] *vs* 80·0 [62·5–87·5]), and core component 1 (IPC programme; 50·0 [13·3–62·9] *vs* 82·5 [72·5–92·5]; table 2; figure 2). The largest differences between public and private facilities were for core component 7 (workload, staffing, and bed occupancy; 60·0 [IQR 39·5–75·0 *vs* 85·0 [70·0–98·0]), core component 4 (HAI surveillance; 67·5 [32·5–85·0] *vs* 87·5 [65·0–95·0]), and core component 5 (multimodal strategies; 60·0 [30·0–80·0] *vs* 80·0 [60·0–90·0]; table 2).

Among health-care facilities with fully completed responses, 15·2% (Nw: 588 of 3873) met all IPCAF indicators designated as WHO IPC minimum requirements; whereas 92·9% (Nw: 3598 of 3873) met at least half of these indicators. This varied by type of facility and income level, ranging from 0% (Nw: 0 of 417) of facilities in low-income countries to 25·6% (278 of 1087) in primary health-care facilities, 9% (24 of 268) in secondary health-care facilities, and 19% (18 of 95) in tertiary health-care facilities in high-income countries (appendix 3 p 32). Among secondary and tertiary health-care facilities with completed surveys for core component 1, 98% (Nw: 944 of 959) reported the presence of an IPC programme. However, fewer of these health-care facilities in low-income countries had access to a full-time IPC professional (14% [Nw: 16 of 116] *vs* 75% [289 of 387]), an allocated IPC budget (16% [18 of 116] *vs* 73% [284 of 387]), and routine microbiological laboratory support (42%; [49 of 116] *vs* 96% [373 of 387]) than in high-income countries. Although most primary, secondary, or tertiary health-care facilities had IPC guidelines for various elements of standard and transmission-based precautions, substantially more secondary and tertiary facilities in high-income than low-income countries had HAI-specific guidelines—for example, prevention of surgical site infection (88% [Nw: 342 of 387 vs 3% [three of 116]) and vascular catheter-associated bloodstream infection (90% [350 of 387] vs 3% [three of 116]). Among primary, secondary, and tertiary facilities with completed surveys for core component 3, fewer low-income countries than high-income countries offered IPC training for health workers (50% [Nw: 213 of 423 *vs* 90% [1613 of 1793]), at least upon hiring, including cleaners or other staff involved in care (39% [167 of 423] *vs* 84% [1498 of 1793]). Although more than 90% (Nw: 350 of 388) of all tertiary care facilities defined surveillance as a component of their IPC programme, only 3% (one of 36) in low-income countries had trained personnel responsible for such activities compared with 99% (106 of 107) in high-income countries. More than 80% of primary, secondary, or tertiary health-care facilities with completed surveys for core component 6 reported monitoring of hand hygiene compliance and trained personnel for such activities, but about half of secondary and tertiary facilities fed back reports to frontline staff (59% [Nw 557: of 952) or leadership (58% [555 of 952]). Among all care levels, fewer low-income countries than high-income countries had a well-defined monitoring plan (18% *vs* 77%). 2061 (59%) of 4378 primary, secondary, or tertiary health-care facilities with completed surveys for core component 7 reported adequate spacing between patient beds, and 2740 (63%) reported a system in place to act on staffing needs. In high-income countries, more than 80% of primary, secondary, and teritary health-care facilities met all minimum requirements for core component 8 on built environment (among those with the respective survey section completed). By comparison, fewer health-care facilities in low-income countries reported functioning hand hygiene stations at all points of care (24% [Nw: 107 of 446]), functioning toilets or latrines (54% [239 of 446), and an energy or power supply (55% [246 of 446]). Additionally, only 68% (302 of 446) of low-income countries had continuously available water services, and 54% (240 of 446) had personal protective equipment.

**Figure 3:**
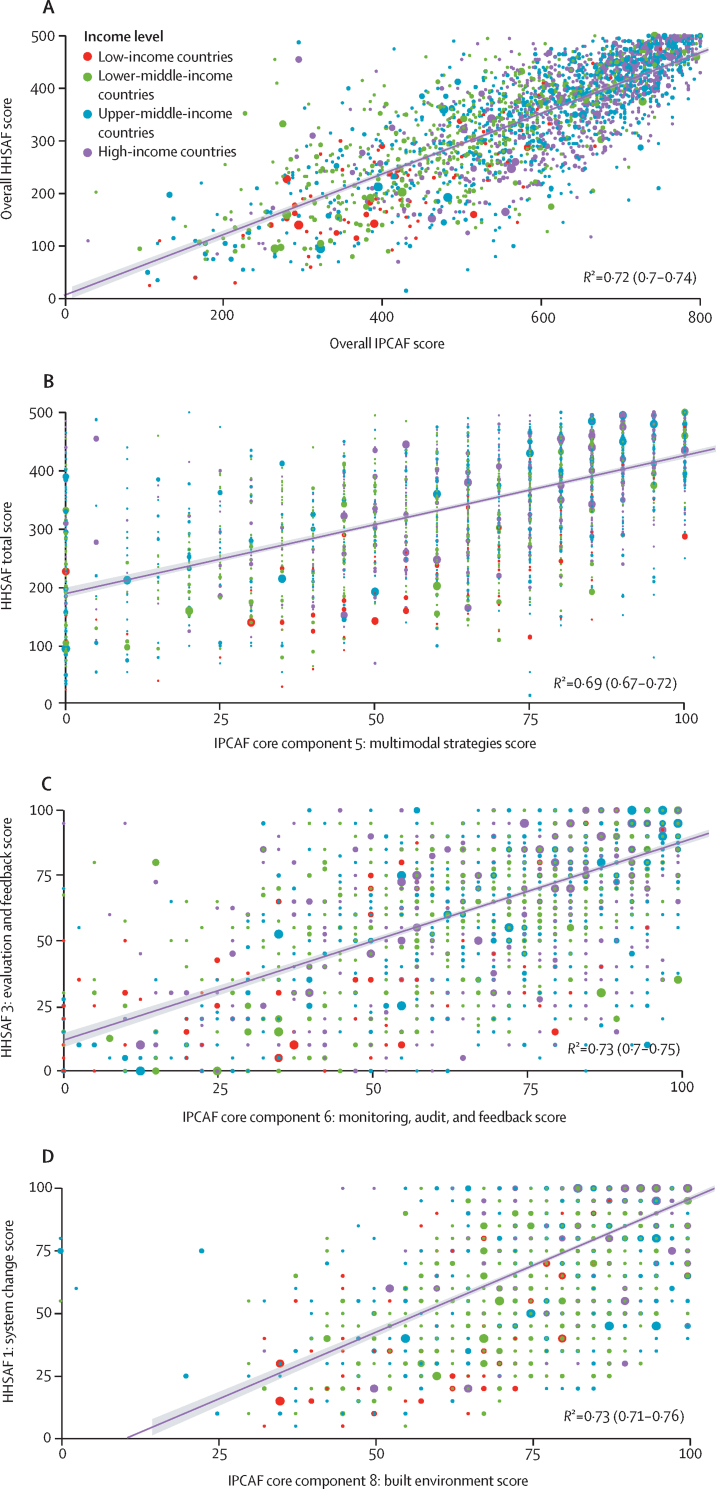
Correlation between weighted median scores for the 2019 HHSAF survey and the IPCAF survey, stratified by World Bank income categories Only includes complete survey responses from health-care facilities that responded to both surveys overall and for the relevant core components.^17^ (A) Correlation between IPCAF and HHSAF total scores (n=2437). (B) Correlation between IPCAF multimodal strategies and HHSAF total scores (n=2543). (C) Correlation between IPCAF monitoring, audit, and feedback and HHSAF evaluation and feedback scores (n=2618). (D) Correlation between IPCAF built environment and HHSAF system scores (n=2593). The size of the coloured dots represent the respective weight. HHSAF=Hand Hygiene Self-Assessment Framework. IPCAF=Infection Prevention and Control Assessment Framework.

The total weighted IPCAF median score positively correlated with the total score calculated for the HHSAF tool (R^2^=0·72; 95% CI 0·70–0·74), which measures the level of progress of hand hygiene implementation (figure 3A).^17^ Specific IPCAF core components positively correlated with the corresponding specific HHSAF elements: core component 5 (multimodal strategies) to the HHSAF total score (R^2^=0·69; 0·67–0·72), core component 6 (audit of IPC practices and feedback) to HHSAF evaluation and feedback (*R*^2^=0·73; 0·70–0·75), and core component 8 (built environment, materials, and equipment for IPC) with HHSAF system change (*R*^2^=0·73; 95% CI 0·71–0·76; figure 3B, C, D).

## Discussion

This global survey provides an important overview of IPC programme implementation in 4440 health-care facilities in 81 countries across all six WHO regions and income levels. To our knowledge, this is the first global assessment using a standardised validated tool providing detailed information about IPC implementation and applying a robust methodology. The total IPCAF score among all health-care facilities corresponded to an advanced level of IPC programme implementation and almost all facilities reported the presence of such a programme. However, only 15·2% of facilities met all indicators considered as minimum requirements for IPC, including none from low-income countries, where, as expected, the level of IPC implementation was significantly lower. Additionally, our survey was combined with a global assessment of hand hygiene programmes, highlighting interesting correlations and further underlining the validity of the use of the IPCAF in the first global IPC survey. IPC implementation strongly correlated with the level of hand hygiene programme implementation, demonstrating alignment between the tools and consistency in health-care facility self-assessment, and emphasising hand hygiene as an important predictor of the overall IPC level.

Overall, the availability of an infrastructure, materials, and equipment for IPC and relevant guidelines were the best implemented components, whereas IPC education and training, adequate workload, staffing, and standards for bed occupancy and spacing scored the lowest. Among low-income countries, HAI surveillance and monitoring and feedback of IPC practices scored the lowest and substantially fewer health-care facilities met built environment minimum requirements. This result is consistent with the 2020 WHO global progress report on WASH in health care facilities,^22^ which documented that only 50% of facilities in least-developed countries had access to water, 37% had access to appropriate sanitation, and 30% had access to waste management services. The present study showed that only a quarter of health-care facilities in low-income countries had access to hand hygiene stations at points of care, and about half had access to functioning toilets, an energy or power supply, water, and personal protective equipment.

Although detailed evaluations of IPC indicators across health-care facilities using a standardised validated tool are rare, four studies in Austria, Germany, Ghana, and Pakistan have used the IPCAF in a comparable manner and reported substantial differences in IPC programme implementation across income levels, similar to our results and to another WHO global IPC cross-sectional study at the national level.^14^, ^15^, ^16^, ^23^, ^24^ In Germany (736 hospitals) and Austria (65 hospitals), results reflected those of high-income countries in our study. In Ghana, the IPCAF was only partly completed to assess IPC preparedness in 56 acute care hospitals, but most facilities scored as intermediate or basic.^14^, ^15^ In Pakistan, the total IPCAF scores among five hospitals in Islamabad scored as inadequate.^23^ In our study, HAI surveillance and monitoring and feedback of IPC practices scored lowest among low-income countries, similar to the study in Pakistan and another study in Georgia among 41 hospitals using an adapted version of the IPCAF.^23^, ^25^

Core components related to workload, staffing, and bed occupancy and IPC education and training scored the lowest across all income and care levels, irrespective of the fact that evidence supporting the WHO guidelines has demonstrated the crucial role of these aspects to reduce pathogen transmission, particularly in outbreak situations.^2^ The COVID-19 pandemic has shown that inadequate staffing and bed capacity can pose huge challenges to country readiness to respond to outbreaks and contribute to their amplification.^26^ Gaps in the effective implementation of IPC training programmes have also been highlighted by other single-country IPCAF surveys.^23^, ^25^ In our study, very few facilities in low-income countries had access to a full-time IPC professional and significantly lower scores were found for core components 1 and 2, indicating defective IPC programmes and limited availability of guidelines. Efforts to build or participate in recognised IPC certification pathways should be strongly encouraged, such as the training programmes offered by the European Committee on Infection Control and the Infection Control African Network. WHO has also defined core competencies for IPC professionals, which can be used as a reference for the development of stronger expertise in countries.^27^

Differences between low-income and high-income countries probably arise from the fact that these activities and systems require regular financial and human resource investments, including a specific expertise, thus highlighting the importance of increased funding dedicated to IPC. In the past decade, a range of investments have been made in low-resource countries to respond to acute needs, such as the recent Ebola epidemics and the COVID-19 pandemic, but the sustainability and ownership of these efforts continue to be questioned.^28^ The systematic lack of data on IPC process and outcome indicators in these countries impedes IPC progress, including the ability to assess the effectiveness of IPC programme implementation, take informed action, and advocate for the importance of IPC (ie, counter the issue of “no data, no problem”). Regular use of the IPCAF and monitoring of hand hygiene practices are effective entry points for improving such data collection. Furthermore, our findings indicate that the IPCAF results for core component 6 (monitoring, audit of IPC practices and feedback) positively correlated with the HHSAF evaluation and feedback. This result shows the relevance of monitoring hand hygiene practices, infrastructures, and other related indicators as a proxy for indicators assessing IPC programmes. We also observed higher IPCAF scores from health-care facilities in countries ranking highest for IPC programme implementation in the TrACSS annual monitoring system, reflecting consistency between these two global self-reporting systems and highlighting the value of tracking progress.

The survey has some limitations. First, we were unable to apply a random sampling approach due to the lack of complete health-care facility registries across all countries. Therefore, selection bias could have occurred, whereby facilities that chose to participate might have had a greater commitment to improving IPC and this could have led to an overestimation of IPCAF scores. Second, although a minimum threshold and post-stratification weights by region and income were applied to improve global representativeness, some country strata had low participation. Third, some facilities did not report their care level, thus limiting the analysis of individual minimum requirements of IPC implementation to a smaller subset. Fourth, training materials and opportunities were provided to enable the correct completion of the survey, but we could not fully support the participation of every facility and those without any IPC experience could have participated and misclassified IPCAF responses. Finally, responses could have also been susceptible to a certain degree of social desirability bias, whereby respondents prefer to select the best answer over the true answer, particularly in countries where data collection was nationally coordinated (although no significant differences were found in this subset).

In conclusion, this robust survey represents the first global overview of IPC programme implementation at the facility level. The findings identify strengths, gaps in IPC implementation, and key opportunities for improvement to inform ongoing global IPC improvement efforts, particularly in low-income and lower middle-income countries, which showed significantly lower IPC implementation. Past and present epidemics have shown how rapidly a few cases of infection by an emerging pathogen in a health-care facility can become a large outbreak due to poor IPC implementation. The endemic burden of HAIs and AMR continues to affect patient safety, hamper high standards of quality of care, and impede the achievement of universal health coverage. To address these challenges and make substantial durable progress in IPC, a greater emphasis must be placed on developing and enforcing stronger policies and regulations, supported by leadership and accountability mechanisms at the highest levels, as well as by an appropriately trained IPC workforce at the facility level.

## Data sharing

The research protocol for this study included a commitment by WHO to restrain from publicly sharing results from individual health-care facilities or results per country to improve participation and minimise social desirability bias. Because aggregated results by WHO region, World Bank income level, hospital type, and level of care are already available in the tables, the main manuscript, and in appendix 3, no other data will be shared.

## Declaration of interests

We declare no competing interests.

## Supplementary Materials

French translation of the abstract

Spanish translation of the abstract
