# Supplementary material for: The first WHO global survey on infection prevention and control in health-care facilities
Source: Lancet Infect Dis. 2022 Jun;22(6):845–56. doi: 10.1016/S1473-3099(21)00809-4 (PMC9132775; doi:10.1016/S1473-3099(21)00809-4)
Supplement: French translation of the abstract [file mmc1.pdf]

THE LANCET
Infectious Diseases

Supplementary appendix 1

This translation in French was submitted by the authors and we reproduce it as supplied. It has not been peer reviewed. *The Lancet*'s editorial processes have only been applied to the original in English, which should serve as reference for this manuscript.

Supplement to: Tomczyk S, Twyman A, de Kraker MEA, et al. The first WHO global survey on infection prevention and control in health-care facilities. *Lancet Infect Dis* 2022; published online Feb 21. https://doi.org/10.1016/ S1473-3099(21)00809-4.

Cette traduction en français a été proposée par les auteurs et nous l'avons reproduite telle quelle. Elle n'a pas été examinée par des pairs. Les processus éditoriaux du *Lancet* n'ont été appliqués qu'à l'original en anglais et c'est cette version qui doit servir de référence pour ce manuscrit.

La première enquête mondiale de l'Organisation Mondiale de la Santé (OMS) sur la prévention et de contrôle des infections dans les établissements de santé

Résumé

Contexte Les composantes principales des programmes de prévention et de contrôle des infections (PCI) établies par l'OMS sont des éléments de base importants pour assurer l'efficacité des programmes de PCI. Nous avons mené la première enquête mondiale de l'OMS pour évaluer la mise en œuvre de ces composantes dans les établissements de santé.

Méthode Dans cette enquête transversale, les professionnels de la PCI ont été invités, par le biais d'une sensibilisation mondiale et d'efforts coordonnés au niveau national, à remplir le modèle pour l'évaluation de la PCI au niveau des établissements de soins de l'OMS en ligne entre le 16 janvier et le 31 décembre 2019. Une pondération post-stratification a été appliquée et les pays dont le taux de réponse était faible ont été exclus de l'enquête pour en améliorer la représentativité. Les scores médians pondérés et les intervalles interquartiles (IIQ) ainsi que les proportions pondérées (Np) répondant aux exigences minimales définies pour les programmes de PCont été rapportés. Les indicateurs associés au score du modèle pour l'évaluation de la PCI ont été évalués au moyen d'une équation d'estimation généralisée.

Résultats Parmi 4440 réponses de 81 pays, le score médian pondéré global du modèle pour l'évaluation de la PCI indiquait un niveau avancé de mise en œuvre (605 [IIQ : 450·4-705·0]), mais des scores significativement plus faibles ont été constatés dans les pays à revenu faible (385, 279·7–442·9) ou intermédiaire de la tranche inférieure (500·4, 345·0–657·5) et dans les établissements publics (515, 385–637·8). Les composantes CP8 (environnement bâti, matériel

et équipement pour la PCI; 90·0, IIQ 75·0–100·0) et CP2 (lignes directrices; 87·5, 70·0–97·5) ont obtenu les scores les plus élevés, et les composantes CP7 (charge de travail, dotation en personnel et occupation des lits; 70·0, 50–90) et CP3 (éducation et formation; 70 ·0, 50·0–85·0) les scores les plus faibles. Dans l'ensemble, seuls 16,8 % (Np : 652/3873) des établissements répondaient à toutes les exigences minimales du modèle pour l'évaluation de la PCI au niveau des établissements de soins, allant de 0 % (Np : 0/417) dans les pays à faible revenu à 25,6 % (Np : 278/1087), 9 % (Np : 24/268) et 18,9 % (Np : 18/95) des établissements de soins primaires/secondaires/tertiaires, respectivement, dans les pays à revenu élevé.

Interprétation Malgré un score PCI globalement élevé à l'échelle mondiale, les importantes lacunes constatées dans la mise en œuvre de la lutte anti-infectieuse au sein des établissements selon les niveaux de revenu et les composantes principales entravent les progrès dans le domaine de la lutte contre les infections. Il est indispensable de soutenir davantage les programmes PCI pour en assurer l'efficacité et la pérennité afin de réduire les risques que représentent les épidémies pour la sécurité sanitaire mondiale et de garantir la sécurité des patients et des agents de santé.

Financement OMS et Programme de PCI, Hôpitaux universitaires de Genève/Faculté de médecine.
