# Supplementary material for: The first WHO global survey on infection prevention and control in health-care facilities
Source: Lancet Infect Dis. 2022 Jun;22(6):845–56. doi: 10.1016/S1473-3099(21)00809-4 (PMC9132775; doi:10.1016/S1473-3099(21)00809-4)
Supplement: Spanish translation of the abstract [file mmc2.pdf]

THE LANCET
Infectious Diseases

Supplementary appendix 2

This translation in Spanish was submitted by the authors and we reproduce it as supplied. It has not been peer reviewed. *The Lancet*'s editorial processes have only been applied to the original in English, which should serve as reference for this manuscript.

Supplement to: Tomczyk S, Twyman A, de Kraker MEA, et al. The first WHO global survey on infection prevention and control in health-care facilities. *Lancet Infect Dis* 2022; published online Feb 21. https://doi.org/10.1016/ S1473-3099(21)00809-4.

Los autores nos proporcionaron esta traducción al español y la reproducimos tal como nos fue entregada. No la hemos revisado. Los procesos editoriales de *The Lancet* se han aplicado únicamente al original en inglés, que debe servir de referencia para este manuscrito.

Primera encuesta mundial de la OMS sobre prevención y control de las infecciones en los establecimientos de salud

Resumen

Antecedentes. Los componentes básicos (CB) de la OMS para la prevención y el control de infecciones (PCI) son elementos importantes para que los programas de PCI sean eficaces. Hasta donde sabemos, llevamos a cabo la primera encuesta mundial de la OMS para evaluar su nivel de puesta en funcionamiento en los establecimientos de salud.

Métodos. En esta encuesta transversal, se invitó a profesionales de PCI a través de actividades de divulgación mundial y esfuerzos nacionales coordinados para que completaran en línea el marco de evaluación de PCI (MEPCI) de la OMS . La encuesta se creó en inglés y luego se tradujo a diez idiomas: alemán, árabe, chino, español, francés, italiano, japonés, ruso, y tailandés.

Se aplicó una ponderación posterior a la estratificación y se excluyeron los países con bajas tasas de respuesta para mejorar la representatividad. Se informó sobre las puntuaciones medias ponderadas y los intervalos intercuartílicos, así como sobre las proporciones ponderadas (Nw por su sigla en inglés) que cumplían los requisitos mínimos definidos en el MEPCI. Los indicadores asociados a la puntuación del MEPCI se evaluaron mediante una ecuación de estimación generalizada.

Resultados. Del 16 de enero al 31 de diciembre de 2019, entre las 4440 respuestas recibidas de 81 países, la puntuación media ponderada total del MEPCI indicaba un nivel de puesta en funcionamiento avanzado (605 [intervalo intercuartílico: 450,4-705]), pero se encontraron puntuaciones mucho más bajas en países de ingresos bajos

(385, 279·7–442·9) y medianos bajos (500·4, 345·0–657·5), y en establecimientos públicos (515, 385–637·8). CB8 «entorno construido, materiales y equipo para la PCI a nivel del establecimiento de atención de la salud » (90; intervalo intercuartílico 75-100) y CB2 «directrices» (87,5; intervalo intercuartílico 70-97,5) obtuvieron la puntuación más alta, y CB7 « carga laboral, dotación de personal y ocupación de camas a nivel de establecimiento de atención de la salud » (70; intervalo intercuartílico 50-90) y CB3 «formación y capacitación» (70; intervalo intercuartílico 50-85) la más baja. En general, solo el 16,8% (proporción ponderada: 652/3873) de los establecimientos cumplía todos los requisitos mínimos del MEPCI, que oscilaban entre el 0% (proporción ponderada: 0/417) en los países de ingresos bajos y el 25,6% (proporción ponderada: 278/1087), el 9% (proporción ponderada: 24/268) y el 18,9% (proporción ponderada: 18/95) de los establecimientos de atención primaria/secundaria/terciaria, respectivamente, en los países de ingresos altos.

Interpretación. A pesar de que la puntuación global del MEPCI es alta, existen notables desigualdades en la puesta en marcha de PCI en los establecimientos en función de los distintos niveles de ingresos y CB, que dificultan el progreso de la PCI. La prestación de un mayor apoyo a programas de PCI más eficaces y sostenibles es fundamental para reducir los riesgos que representan los brotes para la seguridad sanitaria mundial y para garantizar la seguridad de los pacientes y del personal sanitario.

Financiación. OMS y Programa de Control de Infecciones, Hospitales/Facultad de Medicina de la Universidad de Ginebra.
