# Supplementary material for: The first WHO global survey on infection prevention and control in health-care facilities
Source: Lancet Infect Dis. 2022 Jun;22(6):845–56. doi: 10.1016/S1473-3099(21)00809-4 (PMC9132775; doi:10.1016/S1473-3099(21)00809-4)
Supplement: Supplementary appendix 3 [file mmc3.pdf]

THE LANCET
Infectious Diseases

Supplementary appendix 3

This appendix formed part of the original submission and has been peer reviewed. We post it as supplied by the authors.

Supplement to: Tomczyk S, Twyman A, de Kraker MEA, et al. The first WHO global survey on infection prevention and control in health-care facilities. *Lancet Infect Dis* 2022; published online Feb 21. https://doi.org/10.1016/ S1473-3099(21)00809-4.

Supplementary appendix

Content

1) Appendix 1. World Health Organization global survey on infection prevention and control and hand hygiene instructions to participants
2) Appendix 2. Overview of the selection process for the infection prevention and control assessment framework global survey
3) Appendix 3. Approach to survey selection and weighting procedures
4) Appendix 4. Weighted individual infection prevention and control assessment framework (IPCAF) responses determined to be minimum requirements, according to income level, region facility type, and level

Appendix 1. World Health Organization global survey on infection prevention and control and hand hygiene instructions to participants

What is this survey? A WHO global survey on the current status of infection prevention and control (IPC) programmes and hand hygiene activities in health-care facilities, launched within the context of the **WHO annual hand hygiene global campaign (5 May 2019).**

What are the objectives of this survey?

1) To encourage and support local assessments of IPC and hand hygiene activities using standardized and validated tools in the context of the regular work of IPC teams/committees, and the development of local improvement plans in health-care facilities.
2) To conduct a situational analysis on the level of progress of current IPC and hand hygiene activities around the world and inform future efforts and resource use for supporting patient safety, health-care quality improvement, outbreak preparedness and response, and antimicrobial resistance prevention and control.

Timeline: This survey will be open for four months from 16 January to 31 December, 2019.

How the survey works: The survey has two targets involving the completion of two tools at the facility:

1) WHO Infection Prevention and Control Assessment Framework (IPCAF) and
2) WHO Hand Hygiene Self-Assessment Framework (HHSAF).

Both tools are structured, closed-formatted, validated questionnaires with associated scoring systems. The indicators used refer to the recommendations of the *WHO Guidelines on hand hygiene in health care* (https://www.who.int/infection-prevention/publications/hand-hygiene-2009/en/) and the *Core components of infection prevention and control programmes at the national and acute health care facility level* (https://www.who.int/infection-prevention/publications/ipc-components-guidelines/en/). Thus, users should familiarize themselves with these guidelines before completing the tools.

A WHO online system is available for data submission with each tool. English, French, and Spanish versions will be available, as well as some other languages.

Survey enrolment: This survey will be open to any acute health-care facility globally and participation will be voluntary. The WHO IPCAF and HHSAF are facility-level tools and each facility is expected to complete and submit each tool once in the context of this survey. Alternatively, WHO encourages ministries of health (ideally through their national IPC focal point/team) to take the lead in promoting and coordinating the survey process and data collection among health facilities in their country. If any country expresses such an interest, WHO staff can provide additional guidance and establish an agreement ensuring data confidentiality and sharing with national authorities.

Benefits for participants

Facility: Conducting these assessments will help facilities to understand their local situation regarding IPC and hand hygiene using validated and standardized tools. When this process is conducted in the spirit of improvement, it will allow to identify strengths and gaps and greatly inform contextually-relevant IPC plans. By submitting the data via the WHO online survey system, the facility will have the opportunity to be part of a global picture while maintaining data confidentiality and anonymity, and to be provided with automatically generated and downloadable results and scores.

Country: If national authorities decide to actively promote and coordinate the participation of facilities in this survey, they will be able to gather valuable and standardized information om the overall IPC level and status of hand hygiene improvement in the country. This information will highlight where stronger action is needed, whilst contributing to understanding the current situation of IPC and hand hygiene progress globally. On the occasion of this survey, WHO encourages national authorities to also consider undertaking an assessment of the national IPC programme using the national assessment tool IPCAT2 (https://www.who.int/infection-prevention/tools/core-components/en/) in order to gather a comprehensive picture of IPC in the country.

Data use and confidentiality: All data submitted via the WHO online system will be treated as strictly confidential. Each facility will be able to create their own protected account for data submission. Some basic demographic information (Annex 1) about the facility and the person submitting the data in the online system will be requested for security reasons and to facilitate data cleaning and quality checks. Data will be aggregated and analysed anonymously. Thus, it will not be used to assess an individual country or facility's performance and will not be used to inform any regulatory or punitive measures. Data access will be restricted to a trained research team of which all members have signed a confidentiality agreement. If the facility agrees to participate as part of a network coordinated by the ministry of health, data will be confidentially shared with the national authority according to an informed agreement. The protocol of this global survey was reviewed by the WHO ethics review committee and approved with exemption from ethics review.

Who should complete the tools? The IPC lead/focal person (i.e. the individual with adequate IPC expertise) should be appointed to lead the completion of the tools in discussion with the IPC team/committee. A collective and honest discussion to decide upon the answers to select is the best way to take full advantage of this exercise in the spirit of improvement. However, before beginning, it is critical that the facility leadership be informed of the survey objectives, which should be presented as an opportunity to conduct a situational analysis and develop an improvement plan, while avoiding a culture of blame with regards to IPC and patient safety gaps that may be eventually identified.

How to get organized: Given that there are two different tools to complete over a four-month study period, it is suggested that the time be divided in half for each tool as follows:

- Month 1: preparations for IPCAF
- Month 2: IPCAF completion
- Month 3: preparations for HHSAF
- Month 4: HHSAF completion

Completion of each tool may require the involvement of different health-care providers (e.g. IPC team, senior managers, clinical microbiologist, cleaners, etc.). Thus, this timeline should allow for sufficient time and preparation for comprehensive local discussions to ensure the most accurate responses, without impeding normal clinical duties and responsibilities. We suggest completing the tools in a paper version first and then entering the data electronically via the WHO global survey online system.

For further instructions on each individual tool, please see Annexes 2 and 3, and the following links:

1. Specific instructions for the completion of the IPCAF can be found here: http://www.who.int/infection-prevention/tools/core-components/IPCAF-facility.PDF?ua=1
2. Specific instructions for the completion of the HHSAF can be found here: http://www.who.int/gpsc/country_work/hhsa_framework_October_2010.pdf?ua=1

Your participation will be extremely helpful in contributing to global IPC improvement efforts. Thank you for your willingness to consider participation - we sincerely appreciate it! If you have any questions or concerns, please contact info@whoipcsurvey.org.

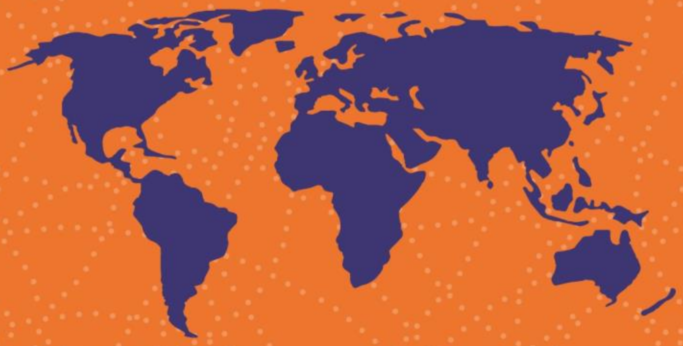

INFECTION PREVENTION AND CONTROL ASSESSMENT FRAMEWORK AT THE FACILITY LEVEL

World Health Organization

Introduction and user instructions

The Infection Prevention and Control (IPC) Assessment Framework (IPCAF) is a tool to support the implementation of the World Health Organization (WHO) *Guidelines on core components of IPC programmes*[1] at the acute health care facility level. The user should be familiar with the contents of these guidelines, including the *Interim practical manual* supporting the implementation of the IPC core components at the facility level[2] before using this tool. The IPCAF is a systematic tool that can provide a baseline assessment of the IPC programme and activities within a health care facility, as well as ongoing evaluations through repeated administration to document progress over time and facilitate improvement.

What is its purpose?

The IPCAF is a structured, closed-formatted questionnaire with an associated scoring system. It is primarily intended to be self-administered (that is, a *self-assessment* tool), but it can also be used for joint assessments, through careful discussions between external assessors (for example, from the Ministry of Health, WHO or other stakeholders) and facility staff. The framework is intended for acute health care facilities, but it can be used in other inpatient health care settings. Although some indicators will be straightforward for high- and middle-income countries, this is a global tool that is valid for assessment of IPC standards in any country. The goal of the framework is to assess the current IPC situation in your facility, that is, existing IPC activities/resources, and identify strengths and gaps that can inform future plans. It can be considered as a diagnostic tool for facilities to detect relevant problems or shortcomings that require improvement and identify areas where they can meet international standards and requirements. If the IPCAF is undertaken as a self-assessment, its usefulness depends on being completed objectively and as accurately as possible. Identifying existing strengths and achievements will help build confidence and convince decision-makers that success and progress is possible. Honestly recognizing gaps will help to create a sense of urgency for the changes needed to improve IPC. For these reasons, it is important to determine the correct score for each section as well as the overall score. Overall, the IPCAF gives a score that can be used as an indicator of the level of progress from an improvement perspective. These results can be used to develop an action plan, using the *Interim practical manual*[2] for the implementation of the IPC core components at the facility level among other resources, to strengthen existing measures and motivate facilities to intensify efforts where needed. By completing it regularly, facilities can monitor their progress over time.

1 WHO Guidelines on core components of IPC programmes at the national and acute health care facility level. 2016 (http://www.who.int/infection-prevention/publications/core-components/en/, accessed 13 April 2018).

2 Improving infection prevention and control at the health facility level. Interim practical manual supporting implementation of the WHO guidelines on core components of infection prevention and control programmes. (http://www.who.int/infection-prevention/tools/core-components/en/, accessed 3 May 2018) WHO/HIS/SDS/2018.9 © World Health Organization 2018. Some rights reserved. This work is available under the CC BY-NC-SA 3.0 IGO.

WHO proposes five steps for the implementation of IPC facility programmes:

1. preparing for action
2. **baseline assessment**
3. developing and executing an action plan
4. **assessing impact**
5. sustaining the programme over the long term.

In particular, the IPCAF is a valuable tool to support Steps 2 and 4 of this process. Step 2 "baseline assessment" is concerned with understanding the current situation, including strengths and weaknesses, to guide action planning for improvement. Step 4 "assessing impact" is concerned with evaluating the effectiveness of activities undertaken in the context of the action plan.

Who should complete and use the IPCAF?

- Health care professionals/teams responsible for organizing and implementing IPC activities, who have in-depth understanding and knowledge of IPC activities at the facility level.
- If there are no professionals in charge of IPC or there is not yet an IPC programme established, the tool should be completed and used by senior facility managers.
- The IPCAF assesses the health care facility as a whole. Of note: in most cases "you" refers to the facility and is not directly addressing the IPC lead/professional answering the question. The IPC team may need to consult with other relevant teams in the facility (for example, health care worker protection and safety, occupational health, surveillance and epidemiology, cleaning and maintenance, environmental health, administration, etc.) to be able to respond to questions accurately.
- The IPCAF is designed for global use at facilities of any size, regardless of their medical focus or development stage.
- If used in joint evaluations, the external assessor should be an IPC professional with an understanding of the recommendations contained in the *WHO Guidelines on core components of IPC programmes*[1].

How is it structured?

The IPCAF is structured according to the recommendations in the *WHO Guidelines on core components of IPC programmes*[1] at the acute health care facility level and thus, it is divided into eight sections reflecting the eight WHO IPC core components, which are then addressed by a total of 81 indicators. These indicators are based on evidence and expert consensus and have been framed as questions with defined answers to provide an orientation for assessment. Based on the overall score achieved in the eight sections, the facility is assigned to one of four levels of IPC promotion and practice.

1. **Inadequate:** IPC core components implementation is deficient. Significant improvement is required.
2. **Basic:** Some aspects of the IPC core components are in place, but not sufficiently implemented. Further improvement is required.
3. **Intermediate:** Most aspects of the IPC core components are appropriately implemented. The facility should continue to improve the scope and quality of implementation and focus on the development of long-term plans to sustain and further promote the existing IPC programme activities.
4. **Advanced:** The IPC core components are fully implemented according to the WHO recommendations and appropriate to the needs of the facility.

How does it work?

When completing the questions contained in the eight sections, choose the answer(s) that most accurately describe(s) the situation at your facility. When you are unfamiliar with terminology in the stated questions, it is strongly recommended to consult the *WHO Guidelines on core components of IPC programmes*[1] or other resources provided in the footnotes to familiarize yourself with new terms and concepts. Difficulties in answering specific questions could indicate that some IPC aspects are not sufficiently developed at your facility and users are encouraged to self-reflect. This can also help lead to improvement. In general, you should choose only one answer per question (questions marked either "yes/no" or "choose one answer"). Some questions are designed to allow multiple answers. These questions are marked with the note "please tick all that apply", which enables you to choose all answers that are appropriate to your facility (choose at least one). Points are allocated to the individual answers of each question, depending on the importance of the question/answer in the context of the respective core component. In each section (core component), a maximum score of 100 points can be achieved. After you have answered all questions of

a component, the score can be calculated by adding the points of every chosen answer. By adding the total scores of all eight components, the overall score is calculated.

Is the IPCAF suitable for inter-facility comparison?

The primary goal of the framework is to provide an orientation to assess the situation of IPC at the individual health care facility level and to monitor the development and improvement of IPC activities over time through repeated use. The comparison of different health care facilities should be done very carefully, particularly when of different sizes, medical focus and socioeconomic setting. Therefore, the framework is not primarily intended for external comparison or benchmarking, but these might be possible - provided that a sound methodology is used.

**Core component 1: Infection Prevention and Control (IPC) programme**		
**Question**	**Answer**	**Score**
**1. Do you have an IPC programme?**[3]<br>Choose one answer	☐ No	0
	☐ Yes, without clearly defined objectives	5
	☐ Yes, with clearly defined objectives and annual activity plan	10
**2. Is the IPC programme supported by an IPC team comprising of IPC professionals?**[4] Choose one answer	☐ No	0
	☐ Not a team, *only* an IPC focal person	5
	☐ Yes	10
**3. Does the IPC team have at least one full-time IPC professional or equivalent (nurse or doctor working 100% in IPC) available?**<br>Choose one answer	☐ No IPC professional available	0
	☐ No, *only* a part-time IPC professional available	2.5
	☐ Yes, one per > 250 beds	5
	☐ Yes, one per ≤ 250 beds	10
**4. Does the IPC team or focal person have dedicated time for IPC activities?**	☐ No	0
	☐ Yes	10
**5. Does the IPC team include both doctors and nurses?**	☐ No	0
	☐ Yes	10
**6. Do you have an IPC committee**[5] **actively supporting the IPC team?**	☐ No	0
	☐ Yes	10

7. Are any of the following professional groups represented/included in the IPC committee?

Senior facility leadership (for example, administrative director, chief executive officer [CEO], medical director)	☐ No	0
	☐ Yes	5
Senior clinical staff (for example, physician, nurse)	☐ No	0

	☐ Yes	2.5
Facility management (for example, biosafety, waste, and those tasked with addressing water, sanitation, and hygiene [WASH])	☐ No	0
	☐ Yes	2.5
**8. Do you have clearly defined IPC objectives (that is, in specific critical areas)?** Choose one answer	☐ No	0
	☐ Yes, IPC objectives *only*	2.5
	☐ Yes, IPC objectives and measurable outcome indicators (that is, adequate measures for improvement)	5
	☐ Yes, IPC objectives, measurable outcome indicators and set future targets	10
**9. Does the senior facility leadership show clear commitment and support for the IPC programme:**		
By an allocated budget specifically for the IPC programme (that is, covering IPC activities, including salaries)?	☐ No	0
	☐ Yes	5
By demonstrable support for IPC objectives and indicators within the facility (for example, at executive level meetings, executive rounds, participation in morbidity and mortality meetings)?	☐ No	0
	☐ Yes	5
**10. Does your facility have microbiological laboratory support (either present on or off site) for routine day-to-day use?** Choose one answer	☐ No	0
	☐ Yes, but not delivering results reliably (timely and of sufficient quality)	5
	☐ Yes, and delivering results reliably (timely and of sufficient quality)	10
**Subtotal score**		**/100**

3 IPC programmes should have clearly defined *objectives* based on local epidemiology and priorities according to risk assessment, and defined *functions and activities* that align with and contribute towards the prevention of health care-associated infections and antimicrobial resistance in health care. They should also include dedicated, trained IPC professionals. See the *WHO Guidelines on core components of IPC programmes at the national and acute health care facility level* for more information (**http://www.who.int/infection-prevention/publications/core-components/en/**, accessed 13 April 2018).

4 IPC professional: medical or nursing staff trained in a certified IPC course.

5 An IPC committee is a multidisciplinary group with interested stakeholders across the facility, which interacts with and advises the IPC team. An IPC team includes dedicated IPC professionals who are responsible for the IPC programme.

Core component 2: Infection Prevention and Control (IPC) guidelines		
**Question**	**Answer**	**Score**
**1. Does your facility have the expertise (in IPC and/or infectious diseases) for developing or adapting guidelines?**	☐ No	0
	☐ Yes	7.5
**2. Does your facility have guidelines available for:**		
Standard precautions?	☐ No	0
	☐ Yes	2.5
Hand hygiene?	☐ No	0
	☐ Yes	2.5
Transmission-based precautions?[6]	☐ No	0
	☐ Yes	2.5
Outbreak management and preparedness?	☐ No	0
	☐ Yes	2.5
Prevention of surgical site infection?[7]	☐ No	0
	☐ Yes	2.5
Prevention of vascular catheter-associated bloodstream infections?	☐ No	0
	☐ Yes	2.5
Prevention of hospital-acquired pneumonia ([HAP]; all types of HAP, including (but not exclusively) ventilator-associated pneumonia)?	☐ No	0
	☐ Yes	2.5
Prevention of catheter-associated urinary tract infections?	☐ No	0
	☐ Yes	2.5
Prevention of transmission of multidrug-resistant (MDR) pathogens?	☐ No	0
	☐ Yes	2.5

Disinfection and sterilization?	☐ No	0
	☐ Yes	2.5
Health care worker protection and safety[8]	☐ No	0
	☐ Yes	2.5
Injection safety?	☐ No	0
	☐ Yes	2.5
Waste management?	☐ No	0
	☐ Yes	2.5
Antibiotic stewardship?[9]	☐ No	0
	☐ Yes	2.5
**3. Are the guidelines in your facility consistent with national/international guidelines (if they exist)?**	☐ No	0
	☐ Yes	10
**4. Is implementation of the guidelines adapted[10] according to the local needs and resources while maintaining key IPC standards?**	☐ No	0
	☐ Yes	10
**5. Are frontline health care workers involved in both planning and executing the implementation of IPC guidelines in addition to IPC personnel?**	☐ No	0
	☐ Yes	10
**6. Are relevant stakeholders (for example, lead doctors and nurses, hospital managers, quality management) involved in the development and adaptation of the IPC guidelines in addition to IPC personnel?**	☐ No	0
	☐ Yes	7.5
**7. Do health care workers receive specific training related to new or updated IPC guidelines introduced in the facility?**	☐ No	0
	☐ Yes	10
**8. Do you regularly monitor the implementation of at least some of the IPC guidelines in your facility?**	☐ No	0
	☐ Yes	10

Subtotal score	/100

6 Transmission-based Precautions are to be used in addition to Standard Precautions for patients who may be infected or colonized with certain infectious agents for which additional precautions are needed to prevent infection transmission. They are based on the routes of transmission of specific pathogens (for example, contact versus droplets). More information can be found in the United States Centers for Disease Control and Prevention Guidelines for Isolation Precautions (**https://www.cdc.gov/infectioncontrol/pdf/guidelines/isolation-guidelines.pdf**, accessed 13 April 2018).

7 If no surgical interventions are undertaken at your facility, choose answer "Yes".

8 Includes aspects of improving working conditions, detection of occupational diseases, health surveillance of workers, pre-employment screening and vaccinations.

9 Refers to the appropriate use of antimicrobials to improve patient outcomes while minimizing the development and spread of resistance. More information can be found in the *WHO Global Framework for Development & Stewardship to Combat Antimicrobial Resistance* (**http://www.who.int/phi/implementation/research/UpdatedRoadmap-Global-Framework-for-Development-Stewardship-tocombatAMR_2017_11_03.pdf?ua=1**, accessed 29 March 2018).

10 IPC team carefully reviews guidelines to prioritize activities according to needs and resources while maintaining key IPC standards.

Core component 3: Infection Prevention and Control (IPC) education and training		
**Question**	**Answer**	**Score**
**1. Are there personnel with the IPC expertise (in IPC and/or infectious diseases) to lead IPC training?**	☐ No	0
	☐ Yes	10
**2. Are there additional non-IPC personnel with adequate skills to serve as trainers and mentors (for example, link nurses or doctors, champions)?** Choose one answer	☐ No	0
	☐ Yes	10
**3. How frequently do health care workers receive training regarding IPC in your facility?** Choose one answer	☐ Never or rarely	0
	☐ New employee orientation *only* for health care workers	5
	☐ New employee orientation and regular (at least annually) IPC training for health care workers offered but not mandatory	10
	☐ New employee orientation and regular (at least annually) mandatory IPC training for all health care workers	15
**4. How frequently do cleaners and other personnel directly involved in patient care receive training regarding IPC in your facility?** Choose one answer	☐ Never or rarely	0
	☐ New employee orientation *only* for other personnel	5
	☐ New employee orientation and regular (at least annually) training for other personnel offered but not mandatory	10
	☐ New employee orientation and regular (at least annually) mandatory IPC training for other personnel	15
**5. Does administrative and managerial staff receive general training regarding IPC in your facility?** Choose one answer	☐ No	0
	☐ Yes	5
**6. How are health care workers and other personnel trained?** Choose one answer	☐ No training available	0
	☐ Using written information and/or oral instruction and/or e-learning *only*	5

	☐ Includes *additional* interactive training sessions (for example, simulation and/or bedside training)	10
**7. Are there periodic evaluations of the effectiveness of training programmes (for example, hand hygiene audits, other checks on knowledge)?** Choose one answer	☐ No	0
	☐ Yes, but not regularly	5
	☐ Yes, regularly (at least annually)	10
**8. Is IPC training integrated in the clinical practice and training of other specialties (for example, training of surgeons involves aspects of IPC)?** Choose one answer	☐ No	0
	☐ Yes, in some disciplines	5
	☐ Yes, in all disciplines	10
**9. Is there specific IPC training for patients or family members to minimize the potential for health care-associated infections (for example, immunosuppressed patients, patients with invasive devices, patients with multidrug-resistant infections)?**	☐ No	0
	☐ Yes	5
**10. Is ongoing development/education offered for IPC staff (for example, by regularly attending conferences, courses)?**	☐ No	0
	☐ Yes	10
**Subtotal score**		**/100**

Core component 4: Health care-associated infection (HAI) surveillance		
**Question**	**Answer**	**Score**
Organization of surveillance		
**1. Is surveillance a defined component of your IPC programme?**	☐ No	0
	☐ Yes	5
**2. Do you have personnel responsible for surveillance activities?**	☐ No	0
	☐ Yes	5
**3. Have the professionals responsible for surveillance activities been trained in basic epidemiology, surveillance and IPC (that is, capacity to oversee surveillance methods, data management and interpretation)?**	☐ No	0
	☐ Yes	5
**4. Do you have informatics/IT support to conduct your surveillance (for example, equipment, mobile technologies, electronic health records)?**	☐ No	0
	☐ Yes	5
Priorities for surveillance - defined according to the scope of care		
**5. Do you go through a prioritization exercise to determine the HAIs to be targeted for surveillance according to the local context (that is, identifying infections that are major causes of morbidity and mortality in the facility)?**[11]	☐ No	0
	☐ Yes	5
**6. In your facility is surveillance conducted for:**		
Surgical site infections?[12]	☐ No	0
	☐ Yes	2.5
Device-associated infections (for example, catheter-associated urinary tract infections, central line-associated bloodstream infections, peripheral-line associated bloodstream infections, ventilator-associated pneumonia)?	☐ No	0
	☐ Yes	2.5
	☐ No	0

Clinically-defined infections (for example, definitions based only on clinical signs or symptoms in the absence of microbiological testing)?	☐ Yes	2.5
Colonization or infections caused by multidrug-resistant[13] pathogens according to your local epidemiological situation?	☐ No	0
	☐ Yes	2.5
Local priority epidemic-prone infections (for example, norovirus, influenza, tuberculosis [TB], severe acute respiratory syndrome [SARS], Ebola, Lassa fever)?	☐ No	0
	☐ Yes	2.5
Infections in vulnerable populations (for example, neonates, intensive care unit, immunocompromised, burn patients)?[14]	☐ No	0
	☐ Yes	2.5
Infections that may affect health care workers in clinical, laboratory, or other settings (for example, hepatitis B or C, human immunodeficiency virus [HIV], influenza)?	☐ No	0
	☐ Yes	2.5
**7. Do you regularly evaluate if your surveillance is in line with the current needs and priorities of your facility?**[11]	☐ No	0
	☐ Yes	5

Methods of surveillance

**8. Do you use reliable surveillance case definitions (defined numerator and denominator according to international definitions [e.g. CDC NHSN/ECDC]**[15] **or if adapted, through an evidence-based adaptation process and expert consultation?**	☐ No	0
	☐ Yes	5
**9. Do you use standardized data collection methods (for example, active prospective surveillance) according to international surveillance protocols (for example, CDC NHSN/ECDC) or if adapted, through an evidence-based adaptation process and expert consultation?**	☐ No	0
	☐ Yes	5
**10. Do you have processes in place to regularly review data quality (for example, assessment of case report forms, review of microbiology results, denominator determination, etc.)?**	☐ No	0
	☐ Yes	5
	☐ No	0

**11. Do you have adequate microbiology and laboratory capacity to support surveillance?**<br>Choose one answer	☐ Yes, can differentiate gram-positive/negative strains but cannot identify pathogens	2.5
	☐ Yes, can reliably identify pathogens (for example, isolate identification) in a timely manner	5
	☐ Yes, can reliably identify pathogens and antimicrobial drug resistance patterns (that is, susceptibilities) in a timely manner	10
**Information analysis and dissemination/data use, linkage, and governance**		
**12. Are surveillance data used to make tailored unit/facility-based plans for the improvement of IPC practices?**	☐ No	0
	☐ Yes	5
**13. Do you analyze antimicrobial drug resistance on a regular basis (for example, quarterly/half-yearly/annually)?**	☐ No	0
	☐ Yes	5
**14. Do you regularly (for example, quarterly/half-yearly/annually) feedback up-to-date surveillance information to:**		
Frontline health care workers (doctors/nurses)?	☐ No	0
	☐ Yes	2.5
Clinical leaders/heads of department	☐ No	0
	☐ Yes	2.5
IPC committee	☐ No	0
	☐ Yes	2.5
Non-clinical management/administration (chief executive officer/chief financial officer)?	☐ No	0
	☐ Yes	2.5
**15. How do you feedback up-to-date surveillance information? (at least annually)**<br>Choose one answer	☐ No feedback	0

	☐ By written/oral information *only*	2.5
	☐ By presentation and interactive problem-orientated solution finding	7.5
**Subtotal score**		**/100**

15 A prioritization exercise should be undertaken to determine which HAIs to target for surveillance according to the local context (for example, areas and/or patients most at risk) according to available resources (see *Interim practical manual* supporting implementation of the WHO Guidelines on Core Components of Infection Prevention and Control Programmes at **http://www.who.int/infectionprevention/tools/core-components/en/**, accessed 3 May 2018)

16 If no surgical interventions are undertaken at your facility, choose answer "Yes".

17 Multidrug-resistant: Non-susceptibility to at least one agent in three or more antimicrobial categories;14 If vulnerable patient populations are not treated at your facility, choose answer "Yes".

18 United States Centers for Disease Control and Prevention (CDC) National Healthcare Safety Network (NHSN) (**https://www.cdc.gov/nhsn/index.html**, accessed 13 April 2018); European Centre for Disease Prevention and Control (ECDC) (**https://ecdc.europa.eu/en/about-us/partnerships-and-networks/disease-and-laboratory-networks/hai-net**, accessed 13 April 2018).

Core component 5: Multimodal strategies[16] for implementation of infection prevention and control (IPC) interventions

Question	Answer	Score
**1. Do you use multimodal strategies[16] to implement IPC interventions?**	☐ No	0
	☐ Yes	15
**2. Do your multimodal strategies include any or all of the following elements:** Choose one answer (the most accurate) per element	**System change**	0
	☐ Element not included in multimodal strategies	0
	☐ Interventions to ensure the necessary infrastructure and continuous availability of supplies are in place	5
	☐ Interventions to ensure the necessary infrastructure and continuous availability of supplies are in place and addressing ergonomics[17] and accessibility, such as the best placement of central venous catheter set and tray	10
	**Education and training**	
	☐ Element not included in multimodal strategies	0
	☐ Written information and/or oral instruction and/or e-learning *only*	5
	☐ *Additional* interactive training sessions (includes simulation and/or bedside training)	10
	**Monitoring and feedback**	
	☐ Element not included in multimodal strategies	0
	☐ Monitoring compliance with process or outcome indicators (for example, audits of hand hygiene or catheter practices)	5
	☐ Monitoring compliance and providing timely feedback of monitoring results to health care workers and key players	10
	**Communications and reminders**	

	☐ Element not included in multimodal strategies	0
	☐ Reminders, posters, or other advocacy/awareness-raising tools to promote the intervention	5
	☐ *Additional* methods/initiatives to improve team communication across units and disciplines (for example, by establishing regular case conferences and feedback rounds)	10
	**Safety climate and culture change**	
	☐ Element not included in multimodal strategies	0
	☐ Managers/leaders show visible support and act as champions and role models, promoting an adaptive approach[18] and strengthening a culture that supports IPC, patient safety and quality	5
	☐ *Additionally*, teams and individuals are empowered so that they perceive ownership of the intervention (for example, by participatory feedback rounds)	10
**3. Is a multidisciplinary team used to implement IPC multimodal strategies?**	☐ No	0
	☐ Yes	15
**4. Do you regularly link to colleagues from quality improvement and patient safety to develop and promote IPC multimodal strategies?**	☐ No	0
	☐ Yes	10
**5. Do these strategies include bundles[19] or checklists?**	☐ No	0
	☐ Yes	10
**Subtotal score**		**/100**

15 See definition at **http://www.who.int/infection-prevention/publications/ipc-cc-mis.pdf?ua=1**, accessed 13 April 2018. The use of multimodal strategies in IPC has been shown to be the best evidence-based approach to achieve sustained system and behavioural change for the implementation of IPC interventions. Multimodal strategy: ≥3 components implemented in an integrated way to achieve improvement of an outcome and change behaviour (for example, hand hygiene practices). Components can include (i) system change (for example, making the necessary infrastructure, supplies and human resources available), (ii) education and training of health care workers and key players (for example, managers), (iii) monitoring infrastructures, practices, processes, outcomes and providing data feedback; (iv) reminders in the workplace/communications; and (v) culture change within the establishment or the strengthening of a safety climate. It also includes tools, such as checklists and bundles, developed by multidisciplinary teams that take into account local conditions. All five areas should be considered and necessary action taken, based on the local context and situation informed by periodic assessments. Lessons from the field of implementation science suggest that targeting only one of these five elements (that is, using a "unimodal" strategy) is more likely to result in improvements that are short-lived and not sustainable. For more information, please see: **http://www.who.int/infection-prevention/publications/ipc-cc-mis.pdf?ua=1**, accessed 13 April 2018 and the Interim practical manual supporting implementation of the WHO Guidelines on Core Components of Infection Prevention and Control Programmes at **http://www.who.int/infection-prevention/tools/core-components/en/**, accessed 3 April 2018.

16 Ergonomics: human factors or an understanding of interactions among humans and elements of a system to optimize human well-being and overall system performance and prevent human error. More information at: http://www.health.org.uk/sites/health/files/IntegratingHumanFactorsWithInfectionAndPreventionControl.pdf, accessed 13 April 2018.

17 Adaptive approaches consider the behavioural, organizational and cultural complexity in health care systems. They aim to improve the local safety climate and motivate local teams to consistently perform best practices by shaping attitudes, beliefs, and values of clinicians. This could include engaging leadership, improving collaborations and team work, and facilitating staff ownership of the intervention. More information at: **https://www.ahrq.gov/professionals/education/curriculum-tools/cusptoolkit/index.html**, accessed 13 April 2018.

18 Bundles: sets of evidence-based practices focused on improving the care process in a structured manner, for example, improvement of catheter insertion. Please note that bundles and multimodal strategies are not the same concept; bundles are tools that can be used to facilitate the implementation of IPC measures, ideally in the context of multimodal strategies[16] which are a much more comprehensive approach.

Core component 6: Monitoring/audit of IPC practices and feedback		
**Question**	**Answer**	**Score**
**1. Do you have trained personnel responsible for monitoring/audit of IPC practices and feedback?**	☐ No	0
	☐ Yes	10
**2. Do you have a well-defined monitoring plan with clear goals, targets and activities (including tools to collect data in a systematic way)?**	☐ No	0
	☐ Yes	7.5
**3. Which processes and indicators do you monitor in your facility?** Tick all that apply	☐ None	0
	☐ Hand hygiene compliance (using the WHO hand hygiene observation tool[20] or equivalent)	5
	☐ Intravascular catheter insertion and/or care	5
	☐ Wound dressing change	5
	☐ Transmission-based precautions and isolation to prevent the spread of multidrug resistant organisms (MDRO)	5
	☐ Cleaning of the ward environment	5
	☐ Disinfection and sterilization of medical equipment/instruments	5
	☐ Consumption/usage of alcohol-based handrub or soap	5
	☐ Consumption/usage of antimicrobial agents	5
	☐ Waste management	5
**4. How frequently is the *WHO Hand Hygiene Self-Assessment Framework Survey*[21] undertaken?** Choose one answer	☐ Never	0
	☐ Periodically, but no regular schedule	2.5
	☐ At least annually	5
**5. Do you feedback auditing reports (for example, feedback on hand hygiene compliance data or other processes) on the state of the IPC activities/performance?** Tick all that apply	☐ No reporting	0
	☐ Yes, within the IPC team	2.5

	☐ Yes, to department leaders and managers in the areas being audited	2.5
	☐ Yes, to frontline health care workers	2.5
	☐ Yes, to the IPC committee or quality of care committees or equivalent	2.5
	☐ Yes, to hospital management and senior administration	2.5
**6. Is the reporting of monitoring data undertaken regularly (at least annually)?**	☐ No	0
	☐ Yes	10
**7. Are monitoring and feedback of IPC processes and indicators performed in a "blame-free" institutional culture aimed at improvement and behavioural change?**	☐ No	0
	☐ Yes	5
**8. Do you assess safety cultural factors in your facility (for example, by using other surveys such as HSOPSC, SAQ, PSCHO, HSC[22])**	☐ No	0
	☐ Yes	5
**Subtotal score**		**/100**

19 WHO hand hygiene monitoring and feedback tools can be found here: **http://www.who.int/infection-prevention/tools/hand-hygiene/evaluation_feedback/en/**, accessed 18 April 2018.

20 *WHO Hand Hygiene Self-Assessment Framework* can be found here: **http://www.who.int/gpsc/country_work/hhsa_framework_October_2010.pdf?ua=1**, accessed 18 April 2018.

21 HSOPSC: Hospital survey on patient safety culture; SAQ: Safety attitudes questionnaire, PSCHO: Patient safety climate in healthcare organizations; HSC: Hospital safety climate scale. A summary of these surveys can be found at: Colla JB, et al. Measuring patient safety climate: a review of survey. Qual Saf Health Care. 2005;14(5):364-6 (**https://www.ncbi.nlm.nih.gov/pubmed/16195571**, accessed 13 April 2018).

Core component 7: Workload, staffing and bed occupancy[23]		
**Question**	**Answer**	**Score**
**Staffing**		
**1. Are appropriate staffing levels assessed in your facility according to patient workload using national standards or a standard staffing needs assessment tool such as the *WHO Workload indicators of staffing need*[24] method?**	☐ No	0
	☐ Yes	5
**2. Is an agreed (that is, WHO or national) ratio of health care workers to patients[25] maintained across your facility?** Choose one answer	☐ No	0
	☐ Yes, for staff in less than 50% of units	5
	☐ Yes, for staff in more than 50% of units	10
	☐ Yes, for all health care workers in the facility	15
**3. Is a system in place in your facility to act on the results of the staffing needs assessments when staffing levels are deemed to be too low?**	☐ No	0
	☐ Yes	10
**Bed occupancy**		
**4. Is the design of wards in your facility in accordance with international standards[26] regarding bed capacity?** Choose one answer	☐ No	0
	☐ Yes, but *only* in certain departments	5
	☐ Yes, for all departments (including emergency department and pediatrics)	15
**5. Is bed occupancy in your facility kept to one patient per bed?** Choose one answer	☐ No	0
	☐ Yes, but *only* in certain departments	5
	☐ Yes, for all units (including emergency departments and pediatrics)	15
**6. Are patients in your facility placed in beds standing in the corridor outside of the room (including beds in the emergency department)?** Choose one answer	☐ Yes, more frequently than twice a week	0
	☐ Yes, less frequently than twice a week	5

	☐ No	15
**7. Is adequate spacing of > 1 meter between patient beds ensured in your facility?**<br>Choose one answer	☐ No	0
	☐ Yes, but *only* in certain departments	5
	☐ Yes, for all departments (including emergency department and pediatrics)	15
**8. Is a system in place in your facility to assess and respond when adequate bed capacity is exceeded?**<br>Choose one answer	☐ No	0
	☐ Yes, this is the responsibility of the head of department	5
	☐ Yes, this is the responsibility of the hospital administration/ management	10
**Subtotal score**		**/100**

22 Particularly for these questions, the IPC team may need to consult with other relevant teams in the facility to be able to respond to questions accordingly.

23 The *WHO Workload indicators of staffing need* method provides health managers with a systematic way to determine how many health workers of a particular type are required to cope with the workload of a given health facility and aid decision-making (**http://www.who.int/hrh/resources/wisn_user_manual/en/**, accessed 13 April 2018).

24 Taking into account all health care workers involved in service delivery and patient care, including clinical staff (doctors, nurses, dentists, medical assistants, etc.), laboratory technicians and other health care workers (for example, cleaners).

25 The *WHO Essential environmental health standards in health care guidance* provides guidance on standards required for health care in medium- and low-resource countries. These guidelines have been written for use by health managers and planners, architects, urban planners, water and sanitation staff, clinical and nursing staff, carers and other health care providers, and health promoters (**http://www.who.int/ water_sanitation_health/publications/ehs_hc/en/**, accessed 13 April 2018).

**Core component 8: Built environment, materials and equipment for IPC at the facility level**[27]		
**Question**	**Answer**	**Score**
**Water**		
**1. Are water services available at all times and of sufficient quantity for all uses (for example, hand washing, drinking, personal hygiene, medical activities, sterilization, decontamination, cleaning and laundry)?** Choose one answer	☐ No, available on average < 5 days per week	0
	☐ Yes, available on average ≥ 5 days per week or every day <u>but</u> not of sufficient quantity	2.5
	☐ Yes, every day <u>and</u> of sufficient quantity	7.5
**2. Is a reliable safe drinking water station present and accessible for staff, patients and families at all times and in all locations/wards?** Choose one answer	☐ No, not available	0
	☐ Sometimes, or only in some places or not available for all users	2.5
	☐ Yes, accessible at all times <u>and</u> for all wards/groups	7.5
**Hand hygiene and sanitation facilities**		
**3. Are functioning hand hygiene stations (that is, alcohol-based handrub solution or soap and water and clean single-use towels) available at all points of care?** Choose one answer	☐ No, not present	0
	☐ Yes, stations present, <u>but</u> supplies are not reliably available	2.5
	☐ Yes, with reliably available supplies	7.5
**4. In your facility, are ≥ 4 toilets <u>or</u> improved latrines**[28] **available for outpatient settings or ≥ 1 per 20 users for inpatient settings?** Choose one answer	☐ Less than required number of toilets or latrines available <u>and</u> functioning	0
	☐ Sufficient number present <u>but</u> not all functioning	2.5
	☐ Sufficient number present <u>and</u> functioning	7.5
**Power supply, ventilation and cleaning**		
**5. In your health care facility, is sufficient energy/power supply available at day <u>and</u> night for all uses (for example, pumping and boiling water, sterilization and decontamination, incineration or alternative treatment technologies, electronic medical devices, general lighting of areas where health care procedures are performed to ensure safe provision of health care and lighting of toilet facilities and showers)?** Choose one answer	☐ No	0
	☐ Yes, sometimes or only in some of the mentioned areas	2.5

	☐ Yes, always and in all mentioned areas	5
**6. Is functioning environmental ventilation (natural or mechanical[29]) available in patient care areas?**	☐ No	0
	☐ Yes	5
**7. For floors and horizontal work surfaces, is there an accessible record of cleaning, signed by the cleaners each day?** Choose one answer	☐ No record of floors and surfaces being cleaned	0
	☐ Record exists, but is not completed and signed daily or is outdated	2.5
	☐ Yes, record completed and signed daily	5
**8. Are appropriate and well-maintained materials for cleaning (for example, detergent, mops, buckets, etc.) available?** Choose one answer	☐ No materials available	0
	☐ Yes, available but not well maintained	2.5
	☐ Yes, available and well-maintained	5
**Patient placement and personal protective equipment (PPE) in health care settings**		
**9. Do you have single patient rooms or rooms for cohorting[30] patients with similar pathogens if the number of isolation rooms is insufficient (for example, TB, measles, cholera, Ebola, SARS)?[31]** Choose one answer	☐ No	0
	☐ No single rooms but rather rooms suitable for patient cohorting available	2.5
	☐ Yes, single rooms are available	7.5
**10. Is PPE[32] available at all times and in sufficient quantity for all uses for all health care workers?** Choose one answer	☐ No	0
	☐ Yes, but not continuously available in sufficient quantities	2.5
	☐ Yes, continuously available in sufficient quantities	7.5
**Medical waste management and sewage**		
**11. Do you have functional waste collection containers for non-infectious (general) waste, infectious waste and, sharps waste in close proximity to all waste generation points?** Choose one answer	☐ No bins or separate sharps disposal	0
	☐ Separate bins present but lids missing or more than 3/4 full; only two bins (instead of three); or bins at some but not all waste generation points	2.5
	☐ Yes	5

**12. Is a functional burial pit/fenced waste dump or municipal pick-up available for disposal of non-infectious (non-hazardous/ general waste)?** Choose one answer	☐ No pit or other disposal method used	0
	☐ Pit in facility but insufficient dimensions; pits/dumps overfilled or not fenced/locked; or irregular municipal waste pick up	2.5
	☐ Yes	5
**13. Is an incinerator or alternative treatment technology for the treatment of infectious and sharp waste (for example, an autoclave) present (either present on or off site and operated by a licensed waste management service), functional and of a sufficient capacity?** Choose one answer	☐ No, none present	0
	☐ Present, but not functional	1
	☐ Yes	5
**14. Is a wastewater treatment system (for example, septic tank followed by drainage pit) present (either on or off site) and functioning reliably?** Choose one answer	☐ No, not present	0
	☐ Yes, but not functioning reliably	2.5
	☐ Yes and functioning reliably	5

Decontamination and sterilization

**15. Does your health care facility provide a dedicated decontamination area and/or sterile supply department (either present on or off site and operated by a licensed decontamination management service) for the decontamination and sterilization of medical devices and other items/equipment?** Choose one answer	☐ No, not present	0
	☐ Yes, but not functioning reliably	2.5
	☐ Yes and functioning reliably	5
**16. Do you reliably have sterile and disinfected equipment ready for use?** Choose one answer	☐ No, available on average < five days per week	0
	☐ Yes, available on average ≥ five days per week or every day, but not of sufficient quantity	2.5
	☐ Yes, available every day and of sufficient quantity	5
**17. Are disposable items available when necessary? (for example, injection safety devices, examination gloves)** Choose one answer	☐ No, not available	0
	☐ Yes, but *only* sometimes available	2.5
	☐ Yes, continuously available	5

**Subtotal score**		**/100**

30 This component can be assessed in more detail using the *WHO Water and sanitation for health facility improvement tool* (WASH FIT) (**http://www.who.int/water_sanitation_health/publications/water-andsanitation-for-health-facility-improvement-tool/en/**, accessed13 April 2018). Particularly for these questions, the IPC team may need to consult with other relevant teams in the facility to be able to respond to questions accordingly and accurately.

31 Improved sanitation facilities include flush toilets into a managed sewer or septic tank and soak-away pit, VIP latrines, pit latrines with slab and composting toilets. To be considered usable, a toilet/latrine should have a door that is unlocked when not in use (or for which a key is available at any time) and can be locked from the inside during use. There should be no major holes or cracks or leaks in the toilet structure, the hole or pit should not be blocked, water should be available for flush/pour flush toilets. It should be within the grounds of the facility and it should be clean as noted by absence of waste, visible dirt and excreta and insects.

32 Natural ventilation: outdoor air driven by natural forces (for example, winds) through building purpose-built openings, including windows, doors, solar chimneys, wind towers and trickle ventilators. Mechanical ventilation: air driven by mechanical vans installed directly in windows or walls or in air ducts for supplying air into, or exhausting air from, a room. More information at: **http://www.who.int/ water_sanitation_health/publications/natural_ventilation/en/**, accessed 13 April 2018.

33 Cohorting strategies should be based on a risk assessment conducted by the IPC team.

34 Negative pressure ventilation conditions in isolation rooms may be necessary to prevention transmission of some organisms (for example, multidrug-resistant TB).

35 Personal Protective Equipment (PPE): Medical non-sterile and surgical sterile gloves, surgical masks, goggles or face shields and gowns are considered as essential PPE. Respirators and aprons should also be available in adequate quantities in all facilities for use when necessary.

Interpretation: A three-step process

1. Add up your points

	Score	
**Section (Core component)**	**Subtotals**	
1. IPC programme		
2. IPC guidelines		
3. IPC education and training		
4. HAI surveillance		
5. Multimodal strategies		
6. Monitoring/audits of IPC practices and feedback		
7. Workload, staffing and bed occupancy		
8. Built environment, materials and equipment for IPC at the facility level		
**Final total score**		**/800**

2. Determine the assigned "IPC level" in your facility using the total score from Step 1

**Total score** (range)	**IPC level**
0–200	Inadequate
201–400	Basic
401–600	Intermediate
601–800	Advanced

3. Review the framework results and develop an action plan

Review the areas identified by this evaluation as requiring improvement in your facility and develop an action plan to address them. To undertake this task, consult the WHO *Interim practical manual* supporting implementation of the WHO Guidelines on Core Components of Infection Prevention and Control Programmes[2] which will provide you with guidance, templates, tips, and examples from around the world as well as with a list of relevant IPC improvement tools. Keep a copy of this assessment to compare with repeated uses in the future.

Appendix 2. Overview of the selection process for the infection prevention and control assessment framework global survey

Figure. Overview of the selection process for the infection prevention and control assessment framework global survey*

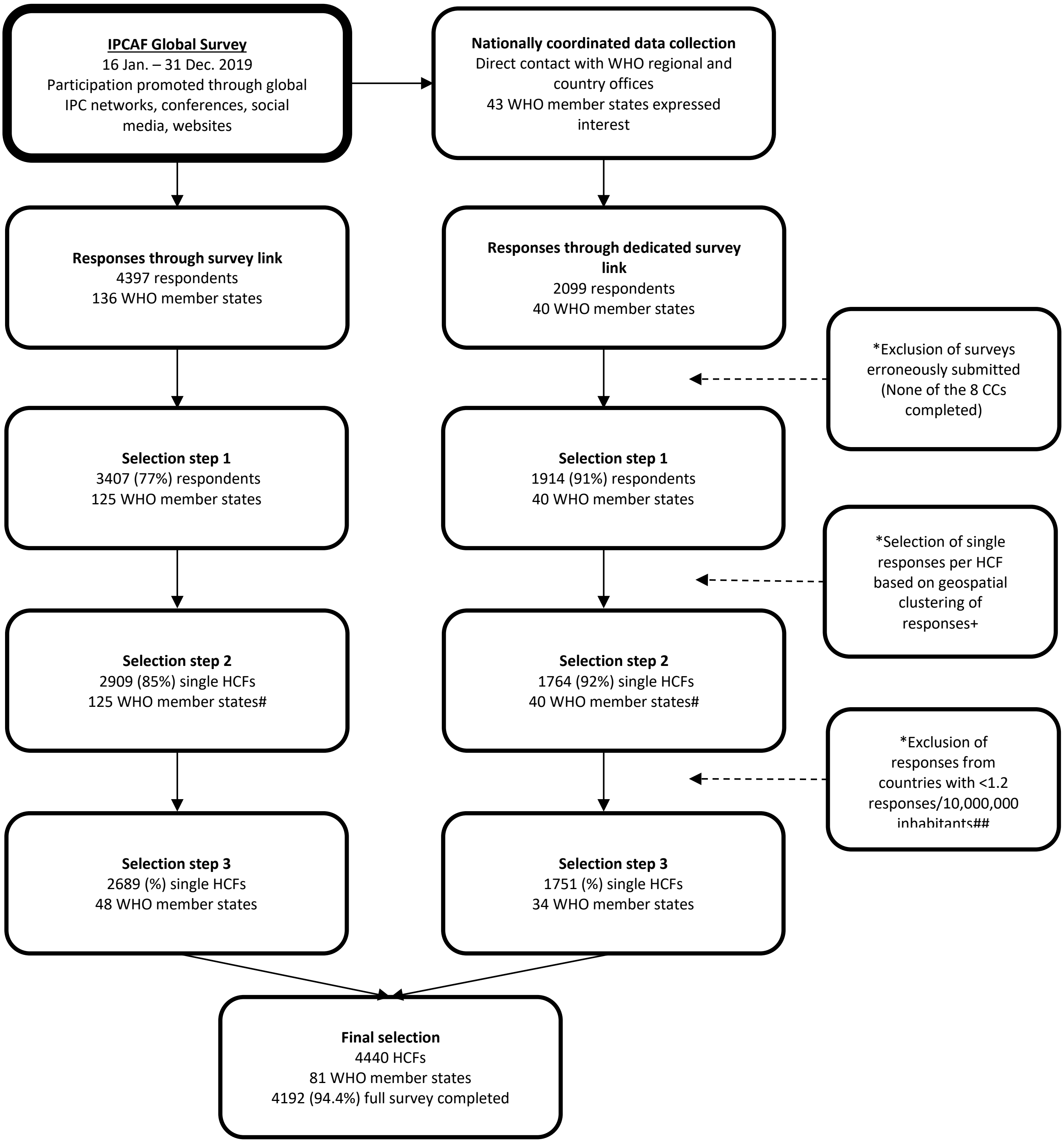

IPCAF=Infection Prevention and Control Assessment Framework; WHO=World Health Organization; HCF=health-care facility; CC=core component.

*Number of selected responses per step and percentages corresponding to the proportion of responses compared to the previous step are shown.

+Participation in the IPCAF global survey was on an individual basis and multiple survey responses could originate from the same health-care facility. To eliminate duplication, responses from the same health-care facility were identified through a geospatial clustering algorithm and a single response per health-care facility was selected through a pre-defined selection strategy.

#HCF in the same member state could either participate individually or part of the national coordinated data collected; overall, prior to the application of the threshold, a total of 4673 unique HCF responses were received from a total of 126 member states.

##The following countries (number of survey responses) were excluded: Afghanistan (n=8); Algeria (n=1); Argentina (n=6); Benin (n=1); Brazil (n=8); Cameroon (n=1); Chad (n=1); China (n=11); Democratic Republic of the Congo (n=9); Czech Republic (n=1); Ethiopia (n=13); Finland (n=1); Guinea (n=1); Hungary (n=1); India (n=14); Indonesia (n=11); Iran (n=21); Israel (n=2); Kenya (n=3); Republic of Korea (n=1); Libya (n=1); Morocco (n=1); Mozambique (n=1); Myanmar (n=6); Nepal (n=4); The Netherlands (n=4); Nigeria (n=33); Pakistan (n=7); Philippines (n=23); Peru (n=2); South Sudan (n=1); Sri Lanka (n=4); Sudan (n=11); Tanzania (n=1); Togo (n=1); Tunisia (n=2); Ukraine (n=1), United States (n=14); Venezuela (n=1).

Appendix 3. Approach to survey selection and weighting procedures

A systematic approach was used to identify and de-duplicate health-care facilities with multiple survey responses from the same facility, namely, a geospatial clustering algorithm was employed based on hospital name, city, country, and a cut-off of 100 metres. The statistical programme R and the packages::functions ‘ggmap::geocode’, ‘SP::SpatialPointsDataFrame’, ‘geosphere::distm’, ‘stats::hclust’, and ‘stats::cutree’ were used (R statistical software version 3.6.1; R Foundation for Statistical Computing, Vienna, Austria). If geospatial clusters were identified, these were manually verified using the following approach: in countries with national campaigns, national focal points were asked to select the response from the facility participating in the national coordinated data collection. Otherwise, the following sequential criteria were used to select the survey response to be retained:

1) record with the most fully completed core component sections
2) record with the most completed individual questions
3) records being part of nationally coordinated data collection, and
4) random selection as needed.

To improve representativeness in the aggregated results, two strategies were used. First, a threshold excluded responses in the analysis from countries with a ratio of the number of survey responses per capita in the lowest ventile. Second, a post-stratification weighting procedure was employed including the following key strata: country, country income level, WHO region, facility level (primary/secondary/tertiary care), and type of facility (private/public) as referenced in the manuscript. To determine weights, a raking algorithm was used with the lowest weight cap possible for convergence. This was done using the statistical programme R ‘anesrake’ package with cap=40. Each country was assigned an equal importance (1/total number of participating countries) and the proportion of countries in each region and income level category determined the overall importance. The marginal distribution for the facility level was based on data from 134 countries reported to the WHO Global Health Observatory in 2013 (primary 72%, secondary 13%, tertiary 10%, other 5%; https://www.who.int/data/gho). No reliable data were found on the distribution of public and private facilities and thus equal weights were selected (private 48%, public 48%, other 4%).

Appendix 4. Weighted individual infection prevention and control assessment framework (IPCAF) responses determined to be minimum requirements according to income level, region facility type, and level

Table 1. Weighted frequencies of facilities meeting all Infection Prevention and Control Assessment Framework (IPCAF) indicator responses that fulfil the 'IPC minimum requirements' according to facility level.

Only includes health-care facilities indicating the level of care (i.e., "Other" level of care was excluded, such as specialized centres or unspecified) and fully completed surveys.

Indicator	All			Low-income			Lower middle-income			Upper middle-income			High-income		
	Nw	Total	%	Nw	Total	%	Nw	Total	%	Nw	Total	%	Nw	Total	%
Primary care facilities	541	2975	18.2	0	301	0	52	588	8.8	211	1000	21.1	278	1087	25.6
Secondary care facilities	28	559	5	0	80	0	0	40	0	4	171	2.3	24	268	9
Tertiary care facilities	19	339	5.6	0	36	0	0	119	0	1	89	1.1	18	95	18.9
All facilities	588	3873	15.2	0	417	0	52	747	7.0	216	1260	17.1	320	1450	22.1

Please note that the following tables are presented separately for primary, secondary and tertiary care facilities compared to the main manuscript text which also presents estimates summarising all facility care levels together or secondary and tertiary levels combined.

Table 2. Weighted frequencies of individual IPCAF indicator responses that fulfil the 'IPC minimum requirements' among primary care facilities.

Only includes health-care facilities indicating a primary level of care and those that completed the respective survey core component section.

Indicator	All		Low-income		Lower-middle-income		Upper-middle-income		High-income	
	Nw	%	Nw	%	Nw	%	Nw	%	Nw	%
**Core component 1: IPC programme**										
None										
**Core component 2: IPC guidelines**	**N=3119**		**N=329**		**N=593**		**N=1017**		**N=1181**	
2. Does your facility have guidelines for standard precautions? Yes	2863	91.8	251	76.3	495	83.5	946	93	1172	99.2
2. Does your facility have guidelines for hand hygiene? Yes	2981	95.6	275	83.6	562	97.8	975	95.9	1169	99.0
2. Does your facility have guidelines for transmission-based precautions? Yes	2714	87	217	66	476	80.3	893	87.8	1127	95.4
2. Does your facility have guidelines for disinfection and sterilization? Yes	2961	94.9	300	91.2	569	96.0	932	91.6	1159	98.1

2. Does your facility have guidelines for health worker protection and safety? Yes	2755	88.3	207	62.9	520	87.8	900	88.5	1128	95.5
2. Does your facility have guidelines for injection safety? Yes	2784	89.3	246	74.8	564	95.1	905	89	1070	90.6
2. Does your facility have guidelines for waste management? Yes	2937	94.2	323	98.2	527	88.9	951	93.5	1137	96.3
8. Do you regularly monitor the implementation of at least some of the IPC guidelines in your facility? Yes	2477	79.4	182	55.3	434	73.2	805	79.2	1056	89.4
**Core component 3: IPC education and training**	**N=3173**		**N=307**		**N=595**		**N=1022**		**N=1250**	
1. Are there personnel with the IPC expertise to lead IPC training? Yes	2454	77.3	193	62.9	411	69.1	756	74	1095	87.6
2. Are there additional non-IPC personnel with adequate skills to serve as trainers and mentors? Yes	2503	78.9	281	91.5	471	79.2	704	68.9	1047	83.8
3. How frequently do health workers receive training regarding IPC in your facility? At least new employee orientation	2773	87.4	176	57.3	461	77.5	908	88.8	1229	98.3
4. How frequently do cleaners and other personnel directly involved in patient care receive training regarding IPC in your facility? At least new employee orientation	2593	81.7	160	52.1	417	70.1	847	82.9	1168	93.4
**Core component 4: Health-care-associated infection surveillance**										
None										
**Core component 5: Multimodal strategies**	**N=3159**		**N=313**		**N=595**		**N=1024**		**N=1227**	
1. Do you use multimodal strategies to implement IPC interventions? Yes	2421	76.6	217	69.3	385	64.7	754	73.6	1065	86.8
**Core component 6: Monitoring/audit and feedback**	3206		329		595		1026		1257	
2. Do you have a well-defined monitoring plan with clear goals, targets and activities? Yes	2031	63.3	50	15.2	324	54.5	678	66.1	979	77.9
3. Which processes and indicators do you monitor in your facility: hand hygiene compliance (using the WHO hand hygiene observation tool or equivalent)? Yes	2667	83.2	217	66	470	79	892	86.9	1089	86.6
**Core component 7: Workload, staffing and bed occupancy**	**N=3185**		**N=330**		**N=592**		**N=1023**		**N=1241**	
2. Is an agreed ratio of health workers to patients maintained across your facility? Yes	2253	70.7%	115	34.8	295	49.8	807	78.9	1036	83.5
3. Is a system in place in your facility to act on the results of the staffing needs assessments when staffing levels are deemed to be too low? Yes	1970	61.9	157	47.6	305	51.5	645	63	864	69.6
**Core component 8: Built environment, materials and equipment**	**N=3161**		**N=329**		**N=592**		**N=1020**		**N=1220**	
1. Are water services available at all times and of sufficient quantity for all uses? Yes, every day and of sufficient quantity	2814	89	223	67.8	462	78	945	92.6	1184	97
3. Are functioning hand hygiene stations available at all points of care? Yes	2166	68.5	60	18.2	285	48.1	802	78.6	1019	83.5
4. In your facility, are >= 4 toilets or improved latrines available for outpatient settings or >= 1 per 20 users for inpatient settings?: Sufficient number present and functioning	2466	78	191	58.1	350	59.1	865	84.8	1060	86.9
5. In your health care facility, is sufficient energy/power supply available at day and night for all uses? Yes	3111	98.4	323	98.2	574	97	994	97.5	1220	100
6. Is functioning environmental ventilation available in patient care areas? Yes	2980	94.3	298	90.6	519	87.7	981	96.2	1182	96.9

9. Do you have single patient rooms or rooms for cohorting patients with similar pathogens if the number of isolation rooms is insufficient? Rooms suitable for patient cohorting or single rooms available	2689	85.1	266	80.9	396	66.9	863	84.6	1165	95.5
10. Is personal protective equipment available at all times and in sufficient quantity for all uses for all health workers? Yes, continuously available in sufficient quantities	2316	73.3	178	54.1	246	41.6	851	83.4	1041	85.3
11. Do you have functional waste collection containers for noninfectious (general) waste, infectious waste and sharps' waste in close proximity to all waste generation points? Yes	2723	86.1	212	64.4	441	74.5	944	92.5	1125	92.2
12. Is a functional burial pit/fenced waste dump or municipal pickup available for disposal of non-infectious (non-hazardous/general waste)? Yes	2508	79.3	198	60.2	383	64.7	872	85.5	1055	86.5
13. Is an incinerator or alternative treatment technology for the treatment of infectious and sharps' waste functional and of a sufficient capacity? Yes	2289	72.4	244	74.2	385	65	714	70	946	77.5
15. Does your health care facility provide a dedicated decontamination area and/or sterile supply department for the decontamination and sterilization of medical devices and other items/equipment? Yes and functioning reliably	2668	84.4	193	58.7	473	79.9	846	82.9	1157	94.8

Table 3. Weighted frequencies of individual IPCAF indicator responses that fulfil the 'IPC minimum requirements' among secondary care facilities.

Only includes health-care facilities indicating a secondary level of care and those that completed the respective survey core component section.

Indicator	All		Low-income		Lower-middle-income		Upper-middle-income		High-income	
	Nw	%	Nw	%	Nw	%	Nw	%	Nw	%
**Core component 1: IPC programme**	**N=574**		**N=80**		**N=41**		**N=171**		**N=283**	
1. Do you have an IPC programme? Yes	559	97.4	79	98.8	40	97.6	170	99.4	270	95.4
3. Does the IPC team have at least one full-time IPC professional or equivalent available? Yes, one per ≤ 250 beds	326	56.8	16	20	2	4.9	94	55	214	75.6
4. Does the IPC team or focal person have dedicated time for IPC activities? Yes	467	81.4	65	81.2	4	9.8	129	75.4	269	95.1
8. Do you have clearly defined IPC objectives (i.e., in specific critical areas)? Yes	464	80.8	78	97.5	5	12.2	163	95.3	218	77
9. Does the senior facility leadership show clear commitment and support for the IPC programme by an allocated budget specifically for the IPC programme? Yes	294	51.2	17	21.2	2	5	75	43.9	200	70.7
**Core component 2: IPC guidelines**	**N=574**		**N=80**		**N=41**		**N=171**		**N=282**	
2. Does your facility have guidelines for standard precautions? Yes	468	81.5	64	80	5	12.2	128	74.9	270	95.7
2. Does your facility have guidelines for hand hygiene? Yes	557	97	65	81.2	40	97.6	170	99.4	282	100
2. Does your facility have guidelines for transmission-based precautions? Yes	432	75.3	34	42.5	5	12.2	124	72.5	270	95.7

2. Does your facility have guidelines for the prevention of surgical site infection? Yes	364	63.4	3	3.8	40	97.6	65	38	256	90.8
2. Does your facility have guidelines for the prevention of vascular catheter-associated bloodstream infections? Yes	320	55.7	3	3.8	5	12.2	56	32.7	257	91.1
2. Does your facility have guidelines for the prevention of hospital-acquired pneumonia? Yes	265	46.2	1	1.2	5	12.2	54	31.6	205	72.7
2. Does your facility have guidelines for the prevention of catheter-associated urinary tract infections? Yes	304	53.0	33	41.2	5	12.2	56	32.7	211	74.8
2. Does your facility have guidelines for disinfection and sterilization? Yes	465	81	64	80	6	14.6	113	66.0	281	99.6
2. Does your facility have guidelines for health worker protection and safety? Yes	454	79.1	64	80	5	12.2	116	67.8	269	95.4
2. Does your facility have guidelines for injection safety? Yes	449	78.2	49	61.3	5	12.2	125	73.1	269	95.4
2. Does your facility have guidelines for waste management? Yes	472	82.2	50	62.5	6	14.6	148	86.5	269	95.4
8. Do you regularly monitor the implementation of at least some of the IPC guidelines in your facility? Yes	399	69.5	63	78.8	5	12.2	121	70.8	210	74.5
**Core component 3: IPC education and training**	**N=574**		**N=80**		**N=41**		**N=171**		**N=283**	
1. Are there personnel with the IPC expertise to lead IPC training? Yes	425	74	63	78.8	5	12.2	132	77.2	225	79.5
2. Are there additional non-IPC personnel with adequate skills to serve as trainers and mentors? Yes	386	67.2	64	80	4	9.8	144	84.2	173	61.1
3. How frequently do health workers receive training regarding IPC in your facility? At least new employee orientation	478	83.3	64	80	5	12.2	126	73.7	283	100
4. How frequently do cleaners and other personnel directly involved in patient care receive training regarding IPC in your facility? At least new employee orientation	461	80.3	64	80	5	12.2	110	64.3	282	99.6
**Core component 4: Health-care-associated infection surveillance**	**N=562**		**N=80**		**N=41**		**N=171**		**N=271**	
1. Is surveillance a defined component of your IPC programme? Yes	421	74.9	2	2.5	5	12.2	156	91.2	258	95.2
**Core component 5: Multimodal strategies**	**N=563**		**N=80**		**N=41**		**N=171**		**N=271**	
1. Do you use multimodal strategies to implement IPC interventions? Yes	366	65	79	98.8	4	9.8	56	32.7	227	83.8
**Core component 6: Monitoring/audit and feedback**	**N=563**		**N=80**		**N=41**		**N=171**		**N=271**	
1. Do you have trained personnel responsible for the monitoring/audit of IPC practices and feedback? Yes	368	65.4	49	61.3	4	9.8	122	71.3	192	70.8
2. Do you have a well-defined monitoring plan with clear goals, targets and activities? Yes	280	49.7	32	40	4	9.8	66	38.6	179	66.1
3. Which processes and indicators do you monitor in your facility: hand hygiene compliance (using the WHO hand hygiene observation tool or equivalent)? Yes	470	83.5	78	97.5	40	97.6	119	69.6	234	86.3
5. Do you feedback auditing reports on the state of the IPC activities/performance? Yes, within the IPC team	389	69.1	78	97.5	4	9.8	113	66.1	194	71.6
5. Do you feedback auditing reports on the state of the IPC activities/performance? Yes, to department leaders and managers in the areas being audited	331	58.8	63	78.8	38	92.7	116	67.8	114	42.1

5. Do you feedback auditing reports on the state of the IPC activities/performance? Yes, to frontline health workers	263	46.7	33	41.2	3	7.3	94	55.0	133	49.1
5. Do you feedback auditing reports on the state of the IPC activities/performance? Yes, to the IPC committee or quality of care committees or equivalent	301	53.5	17	21.2	3	7.3	99	57.9	182	67.2
5. Do you feedback auditing reports on the state of the IPC activities/performance? Yes, to hospital management and senior administration	287	51	18	22.5	39	95.1	91	53.2	140	51.7
**Core component 7: Workload, staffing and bed occupancy**	**N=563**		**N=80**		**N=41**		**N=171**		**N=271**	
2. Is an agreed ratio of health workers to patients maintained across your facility? Yes	423	75.1	62	77.5	4	9.8	114	66.7	244	90
3. Is a system in place in your facility to act on the results of the staffing needs assessments when staffing levels are deemed to be too low? Yes	336	59.7	34	42.5	38	92.7	70	40.9	195	72
7. Is adequate spacing of > 1 metre between patient beds ensured in your facility? Yes, for all departments (including emergency department and paediatrics)? Yes	327	58.1	63	78.8	2	4.9	94	55	168	62
8. Is a system in place in your facility to assess and respond when adequate bed capacity is exceeded? Yes, this is the responsibility of the hospital administration/management	349	62	63	78.8	4	9.8	78	45.6	204	75.3
**Core component 8: Built environment, materials and equipment**	**N=562**		**N=80**		**N=41**		**N=171**		**N=271**	
1. Are water services available at all times and of sufficient quantity for all uses? Yes, every day and of sufficient quantity	519	92.3	78	97.5	40	97.6	131	76.6	270	99.6
3. Are functioning hand hygiene stations available at all points of care? Yes	386	68.7	47	58.8	2	4.9	93	54.4	244	90
4. In your facility, are >= 4 toilets or improved latrines available for outpatient settings or >= 1 per 20 users for inpatient settings? Yes, sufficient number present and functioning	429	76.3	48	60	4	9.8	122	71.3	256	94.5
5. In your health care facility, is sufficient energy/power supply available at day and night for all uses? Yes, always and in all mentioned areas	516	91.8	48	60	39	95.1	159	93	271	100
6. Is functioning environmental ventilation available in patient care areas? Yes	553	98.4	79	98.8	40	97.6	164	95.9	271	100
9. Do you have single patient rooms or rooms for cohorting patients with similar pathogens if the number of isolation rooms is insufficient? Yes, rooms suitable for patient cohorting or single rooms available	557	99.1	80	100	40	97.6	167	97.7	270	99.6
10. Is personal protective equipment available at all times and in sufficient quantity for all uses for all health workers? Yes, continuously available in sufficient quantities	440	78.3	62	77.5	38	92.7	96	56.1	243	89.7
11. Do you have functional waste collection containers for noninfectious (general) waste, infectious waste and, sharps' waste in close proximity to all waste generation points? Yes	471	83.8	63	78.8	5	12.2	133	77.8	270	99.6
12. Is a functional burial pit/fenced waste dump or municipal pickup available for disposal of non-infectious (non-hazardous/general waste)? Yes	430	76.5	48	60	4	9.8	119	69.6	259	95.6
13. Is an incinerator or alternative treatment technology for the treatment of infectious and sharps' waste functional and of a sufficient capacity? Yes	469	83.5	80	100	3	7.3	142	83	245	90.4

15. Does your health care facility provide a dedicated decontamination area and/or sterile supply department for the decontamination and sterilization of medical devices and other items/equipment? Yes, and functioning reliably	556	98.9	77	96.2	39	95.1	169	98.8	271	100

Table 4. Weighted frequencies of individual IPCAF indicator responses that fulfil the 'IPC minimum requirements' among tertiary care facilities.

Only includes health-care facilities indicating a tertiary level of care and those that completed the respective survey core component section.

**Indicator**	**All**		**Low-income**		**Lower-middle-income**		**Upper-middle-income**		**High-income**	
	**Nw**	**%**	**Nw**	**%**	**Nw**	**%**	**Nw**	**%**	**Nw**	**%**
**Core component 1: IPC programme**	**N=385**		**N=36**		**N=120**		**N=126**		**N=104**	
1. Do you have an IPC programme? Yes	385	100	36	100	119	99.2	126	100	104	10
3. Does the IPC team have at least one full-time IPC professional or equivalent available? Yes, one per ≤ 250 beds	180	46.8	0	0	3	2.5	103	81.7	75	72.1
4. Does the IPC team or focal person have dedicated time for IPC activities? Yes	347	90.1	1	2.8	119	99.2	124	98.4	103	99
6. Do you have an IPC committee actively supporting the IPC team? Yes	314	81.6	1	2.8	83	69.2	126	100	104	100
8. Do you have clearly defined IPC objectives (i.e., in specific critical areas)? Yes	362	94.0	36	100	104	86.7	125	99.2	97	93.3
9. Does the senior facility leadership show clear commitment and support for the IPC programme by an allocated budget specifically for the IPC programme? Yes	268	69.6	0	0	117	97.5	67	53.2	83	79.8
10. Does your facility have microbiological laboratory support for routine day-to-day use? Yes	350	90.9	1	2.8	119	99.2	126	100	104	100
**Core component 2: IPC guidelines**	**N=386**		**N=36**		**N=119**		**N=127**		**N=104**	
2. Does your facility have guidelines for standard precautions? Yes	384	99.5	36	100	119	100	126	99.2	103	99
2. Does your facility have guidelines for hand hygiene? Yes	349	90.4	36	100	119	100	91	71.7	103	99
2. Does your facility have guidelines for transmission-based precautions? Yes	366	94.8	35	97.2	103	86.6	124	97.6	103	99
2. Does your facility have guidelines for the prevention of surgical site infection? Yes	227	58.8	0	0	53	44.5	88	69.3	85	81.7

2. Does your facility have guidelines for the prevention of vascular catheter-associated bloodstream infections? Yes	270	69.9	0	0	88	73.9	89	70.1	93	89.4
2. Does your facility have guidelines for the prevention of hospital-acquired pneumonia? Yes	291	75.4	35	97.2	83	69.7	88	69.3	85	81.7
2. Does your facility have guidelines for the prevention of catheter-associated urinary tract infections? Yes	282	73.1	35	97.2	68	57.1	89	70.1	89	85.6
2. Does your facility have guidelines for the prevention of transmission of multidrug-resistant pathogens? Yes	303	78.5	35	97.2	52	43.7	123	96.9	93	89.4
2. Does your facility have guidelines for disinfection and sterilization? Yes	349	90.4	36	100	84	70.6	125	98.4	104	100
2. Does your facility have guidelines for health worker protection and safety? Yes	349	90.4	36	100	88	73.9	125	98.4	100	96.2
2. Does your facility have guidelines for injection safety? Yes	340	88.1	36	100	119	100	86	67.7	100	96.2
2. Does your facility have guidelines for waste management? Yes	346	89.6	36	100	84	70.6	126	99.2	100	96.2
8. Do you regularly monitor the implementation of at least some of the IPC guidelines in your facility? Yes	339	87.8	0	0	119	100	124	97.6	96	92.3
**Core component 3: IPC education and training**	**N=392**		**N=36**		**N=119**		**N=126**		**N=110**	
1. Are there personnel with the IPC expertise to lead IPC training? Yes	339	86.5	36	100	84	70.6	113	89.7	107	97.2
2. Are there additional non-IPC personnel with adequate skills to serve as trainers and mentors? Yes	301	76.8	36	100	118	99.2	79	62.7	68	61.8
3. How frequently do health workers receive training regarding IPC in your facility? New employee orientation and regular (at least annually) training for other personnel offered or mandatory	331	84.4	1	2.8	118	99.2	110	87.3	102	92.7
4. How frequently do cleaners and other personnel directly involved in patient care receive training regarding IPC in your facility? New employee orientation and regular (at least annually) training for other personnel offered or mandatory	285	72.7	0	0	83	69.7	110	87.3	91	82.7
**Core component 4: Health care-associated surveillance**	**N=388**		**N=36**		**N=120**		**N=126**		**N=107**	
1. Is surveillance a defined component of your IPC programme? Yes	350	90.2	36	100	84	70	125	99.2	106	99.1

2. Do you have personnel responsible for surveillance activities? Yes	315	81.2	1	2.8	84	70	125	99.2	106	99.1
3. Have the professionals responsible for surveillance activities been trained in basic epidemiology, surveillance and IPC? Yes	252	64.9	1	2.8	83	69.2	70	55.6	98	91.6
14. Do you regularly feedback up-to-date surveillance information to? Frontline health workers	273	70.4	0	0	82	68.3	111	88.1	80	74.8
14. Do you regularly feedback up-to-date surveillance information to? Clinical leaders/heads of department	279	71.9	0	0	83	69.2	112	88.9	84	78.5
14. Do you regularly feedback up-to-date surveillance information to? IPC committee	337	86.9	0	0	117	97.5	114	90.5	105	98.1
14. Do you regularly feedback up-to-date surveillance information to? Non-clinical management/administration	243	62.6	0	0	81	67.5	104	82.5	58	54.2
**Core component 5: Multimodal strategies**	**N=388**		**N=36**		**N=119**		**N=125**		**N=108**	
1. Do you use multimodal strategies to implement IPC interventions? Yes	305	78.6	36	100	88	73.9	82	65.6	100	92.6
**Core component 6: Monitoring/audit and feedback**	**N=390**		**N=36**		**N=119**		**N=125**		**N=111**	
1. Do you have trained personnel responsible for monitoring/audit of IPC practices and feedback? Yes	372	95.4	36	100	118	99.2	123	98.4	96	86.5
2. Do you have a well-defined monitoring plan with clear goals, targets and activities? Yes	269	69	0	0	52	43.7	122	97.6	95	85.6
3. Which processes and indicators do you monitor in your facility: hand hygiene compliance (using the WHO hand hygiene observation tool or equivalent)? Yes	330	84.6	1	2.8	103	86.6	124	99.2	103	92.8
5. Do you feedback auditing reports on the state of the IPC activities/performance? Yes, within the IPC team	357	91.5	35	97.2	118	99.1	114	91.2	90	81.1
5. Do you feedback auditing reports on the state of the IPC activities/performance) Yes, to department leaders and managers in the areas being audited	328	84.1	1	2.8	117	98.3	122	97.6	89	80.2
5. Do you feedback auditing reports on the state of the IPC activities/performance? Yes, to frontline health workers	294	75.4	0	0	116	97.5	106	84.8	72	64.9
5. Do you feedback auditing reports on the state of the IPC activities/performance? Yes, to the IPC committee or quality of care committees or equivalent	289	74.1	0	0	117	98.3	89	71.2	83	74.8

5. Do you feedback auditing reports on the state of the IPC activities/performance? Yes, to hospital management and senior administration	268	68.7	0	0	102	85.7	86	68.8	80	72.1
**Core component 7: Workload, staffing and bed occupancy**	**N=390**		**N=36**		**N=119**		**N=125**		**N=111**	
2. Is an agreed ratio of health workers to patients maintained across your facility? Yes	243	62.3	0	0	67	56.3	81	64.8	95	85.6
3. Is a system in place in your facility to act on the results of the staffing needs assessments when staffing levels are deemed to be too low? Yes	241	61.8	1	2.8	101	84.9	70	56	69	62.2
7. Is adequate spacing of >1 metre between patient beds ensured in your facility? Yes, for all departments (including emergency department and paediatrics)	188	48.2	0	0	42	35.3	74	59.2	72	64.9
**Core component 8: Built environment, materials and equipment**	**N=355**		**N=36**		**N=119**		**N=90**		**N=110**	
1. Are water services available at all times and of sufficient quantity for all uses? Yes, every day and of sufficient quantity	283	79.7	0	0	83	69.7	89	98.9	110	100
3. Are functioning hand hygiene stations available at all points of care? Yes	260	73.2	0	0	81	68.1	84	93.3	95	86.4
4. In your facility, are >= 4 toilets or improved latrines available for outpatient settings or >= 1 per 20 users for inpatient settings? Yes, sufficient number present and functioning	262	73.8	0	0	81	68.1	85	94.4	95	86.4
5. In your health care facility, is sufficient energy/power supply available at day and night for all uses? Yes, always and in all mentioned areas	287	80.8	1	2.8	118	99.2	58	64.4	110	100
6. Is functioning environmental ventilation available in patient care areas? Yes	353	99.4	36	100	119	100	88	97.8	110	100
9. Do you have single patient rooms or rooms for cohorting patients with similar pathogens if the number of isolation rooms is insufficient? Yes, rooms suitable for patient cohorting or single rooms available	318	89.6	1	2.8	118	99.2	90	100	110	100
10. Is personal protective equipment available at all times and in sufficient quantity for all uses for all health workers? Yes, continuously available in sufficient quantities	291	82	0	0	116	97.5	76	84.4	99	90

11. Do you have functional waste collection containers for noninfectious (general) waste, infectious waste and, sharps' waste in close proximity to all waste generation points? Yes	282	79.4	0	0	82	68.9	90	100	110	100
12. Is a functional burial pit/fenced waste dump or municipal pickup available for disposal of non-infectious (non-hazardous/general waste)? Yes	278	78.3	0	0	83	69.7	89	98.9	106	100
13. Is an incinerator or alternative treatment technology for the treatment of infectious and sharps' waste functional and of a sufficient capacity? Yes	299	84.2	1	2.8	118	99.2	86	95.6	95	86.4
15. Does your health-care facility provide a dedicated decontamination area and/or sterile supply department for the decontamination and sterilization of medical devices and other items/equipment? Yes and functioning reliably	347	97.7	36	100	118	99.2	90	100	104	94.5
